# A regression based approach to phylogenetic reconstruction from multi-sample bulk DNA sequencing of tumors

**DOI:** 10.1371/journal.pcbi.1012631

**Published:** 2024-12-04

**Authors:** Henri Schmidt, Benjamin J. Raphael

**Affiliations:** Department of Computer Science, Princeton University, New Jersey, United States of America; National Library of Medicine, UNITED STATES OF AMERICA

## Abstract

**Motivation:**

DNA sequencing of multiple bulk samples from a tumor provides the opportunity to investigate tumor heterogeneity and reconstruct a phylogeny of a patient’s cancer. However, since bulk DNA sequencing of tumor tissue measures thousands of cells from a heterogeneous mixture of distinct sub-populations, accurate reconstruction of the tumor phylogeny requires simultaneous deconvolution of cancer clones and inference of ancestral relationships, leading to a challenging computational problem. Many existing methods for phylogenetic reconstruction from bulk sequencing data do not scale to large datasets, such as recent datasets containing upwards of ninety samples with dozens of distinct sub-populations.

**Results:**

We develop an approach to reconstruct phylogenetic trees from multi-sample bulk DNA sequencing data by separating the reconstruction problem into two parts: a structured regression problem for a fixed tree T, and an optimization over tree space. We derive an algorithm for the regression sub-problem by exploiting the unique, combinatorial structure of the matrices appearing within the problem. This algorithm has both asymptotic and empirical improvements over linear programming (LP) approaches to the problem. Using our algorithm for this regression sub-problem, we develop *fastBE*, a simple method for phylogenetic inference from multi-sample bulk DNA sequencing data. We demonstrate on simulated data with hundreds of samples and upwards of a thousand distinct sub-populations that *fastBE* outperforms existing approaches in terms of reconstruction accuracy, sample efficiency, and runtime. Owing to its scalability, *fastBE* enables both phylogenetic reconstruction directly from indvidual mutations without requiring the clustering of mutations into clones, as well as a new phylogeny constrained mutation clustering algorithm. On real data from fourteen B-progenitor acute lymphoblastic leukemia patients, *fastBE* infers mutation phylogenies with fewer violations of a widely used evolutionary constraint and better agreement to the observed mutational frequencies. Using our phylogeny constrained mutation clustering algorithm, we also find mutation clusters with lower distortion compared to state-of-the-art approaches. Finally, we show that on two patient-derived colorectal cancer models, *fastBE* infers mutation phylogenies with less violation of a widely used evolutionary constraint compared to existing methods.

## Author summary

DNA sequencing of a bulk tumor sample measures the genomes of the heterogeneous mixture of cells that comprise a tumor. Reconstructing the evolutionary history of a cancer from such admixed measurements is challenging, as standard phylogenetic techniques assume that genomes of individual cells are measured. Multiple specialized techniques aim to simultaneously infer the unmeasured genomes and construct the evolutionary history of these genomes, but many of these methods do not scale to large numbers of genomes in the mixture. We introduce a new tool, fast Bulk Evolution (*fastBE*), which accurately reconstructs the evolutionary history of tumors containing hundreds-thousands of genomes from bulk DNA sequencing data. Key to the success of *fastBE* are new algorithmic insights which make this task tractable. *fastBE* is a useful tool to analyze large multi-region tumor sequencing datasets.

## 1 Introduction

Tumor evolution is characterized by the accumulation of somatic genomic alterations that alter the fitness of sub-populations of cells, leading to unregulated growth. Over the past ten years, high-coverage DNA sequencing of bulk tumor samples has proven tremendously successful in deciphering this complex evolutionary process [1–3]. There are now dozens of computational techniques [4–12] to accurately and efficiently identify distinct sub-populations of cells in a tumor sample and reconstruct the evolutionary history, or a phylogeny, of these populations. Application of these techniques can help identify the genomic alterations that drive tumor growth [13, 14]. Recent studies have demonstrated that intra-tumor heterogeneity is more prevalent than previously reported (reviewed in [15]). For example, the TracerX Consortium [2] found up to fifteen distinct sub-populations of cells, or *subclones*, from multi-region sequencing of a patient biopsy of non-small-cell lung cancer. Further, they noticed that without this multi-region sequencing, up to 65% of subclones and 76% of subclonal mutations would have been missed, suggesting that perhaps further heterogeneity could be uncovered by increasing the number of sequenced samples. In tandem with decreasing sequencing costs, high levels of intra-tumor heterogeneity have led to bulk-DNA sequencing datasets with an increasingly large number of samples and subclones—containing up to 90 samples and 26 subclones for a single cancer patient [16].

While numerous methods have been developed to build phylogenies from bulk DNA sequencing of tumors, few of these methods scale to datasets with dozens of samples and subclones from the same patient. Recently [11] demonstrated that existing methods fail to scale beyond even ten subclones, making their application to datasets with high amounts of intra-tumor heterogeneity challenging. As another consequence of poor scalability, all existing methods—except the newly introduced method Orchard [12]—infer phylogenies using summary statistics computed from *clusters of mutations* rather than the individual mutation read counts, potentially missing valuable phylogenetic signal.

**Contribution.** In this work, we describe a *structured regression* formulation of the successful matrix factorization model [5–7, 9, 10] for phylogenetic inference from somatic single nucleotide variants (SNVs) measured via DNA sequencing of one or more bulk tumor samples from the same patient. In particular, we identify a tractable, *ℓ*_1_-regression problem hidden within the NP-complete, variant allele frequency (VAF) factorization problem [5, 17]—analogous to the *method of minimum evolution* in species phylogenetics [18, 19]—where a tractable regression sub-problem [20] is solved within an NP-complete [21, 22] optimization problem. By studying the unique, combinatorial *structure* of the clonal matrices [5] appearing within this *ℓ*_1_-regression sub-problem, we derive an algorithm which obtains both asymptotic and empirical improvements over a naïve, linear programming based approach. Further, our regression algorithm efficiently recomputes the solution to the *ℓ*_1_-regression sub-problem upon slight modifications to the tree topology, such as the addition of vertices and subtree prune-and-regraft (SPR) operations [23].

Utilizing our fast *ℓ*_1_-regression algorithm and incorporating recently introduced combinatorial search techniques [12], we develop a simple method, *fastBE* (*fast* *B*ulk *E*volution), for phylogenetic inference from multi-sample bulk DNA sequencing data. We show that on simulated data, *fastBE* outperforms existing methods for inferring the ground truth phylogeny across a variety of metrics—including sample efficiency—while running orders of magnitude faster. For example, *fastBE* solves simulated instances with up to 1000 clones and 100 samples in under half an hour. Building on the scalability of *fastBE*, we develop a phylogeny constrained mutation clustering algorithm which clusters mutations using a mutation phylogeny inferred by *fastBE* and outperforms state-of-the-art mutation clustering methods. Applying *fastBE* to a multi-sample dataset from fourteen patients with acute lymphoblastic leukemias [16], we show that our inferred mutation phylogenies are similar to those inferred by Pairtree [11] and Orchard [12], but better recapitulate observed mutational frequencies and possess fewer violations of the *sum condition* [5–7, 24]. Finally, we demonstrate that on two patient-derived colorectal cancer models *fastBE* finds mutation phylogenies with fewer violations of the sum condition compared to existing methods.

## 2 Materials and methods

### 2.1 Background and related work

Following previous work, we restrict attention to somatic, single-nucleotide mutations in copy neutral regions as phylogenetic characters of cancer evolution. We assume that each *genomic locus* is mutated exactly once during this evolutionary process, known as the *infinite sites assumption* [5–11]. The problem of inferring a phylogeny from DNA sequencing data from multiple bulk samples then corresponds to a matrix factorization problem—called the variant allele frequency factorization problem (VAFFP) [5]—which we describe below. For extending this model under violations of the infinite sites assumption, see Sections B.5, B.6 in S1 Text.

Under these assumptions, the evolutionary history of a tumor is described as a rooted, phylogenetic tree T=(V(T),E(T)) where the vertices *V* correspond to sub-populations of cells containing identical sets of mutations, or *clones*, and the edges *E* define ancestral relationships between the clones. Mathematically, a mutation is represented by its position *j* in the genome and a clone *b*_*i*_ is a length *n* binary vector where *b*_*i*,*j*_ = 1 (resp. *b*_*i*,*j*_ = 0) denotes the presence (resp. absence) of mutation *j* in the *i*^th^ clone. As each mutation occurs exactly once during the evolutionary process, there are *n* distinct clones *b*_*i*_, and they form an important subclass of *perfect phylogenies* [25, 26] where the internal vertices, in addition to the leaves, are labeled [5, 27]. Then, the evolutionary history of a tumor is given by a vertex labeled, perfect phylogeny with *n* vertices, which we call an *n-clonal tree* to emphasize that each of the *n* vertices correspond to a distinct tumor clone.

In practice, rather than modeling the evolution of individual mutations, mutations are typically clustered into mutation clusters using tools such as PyClone [28, 29] or SciClone [30] to make inference computationally tractable. These clusters of mutations are then assumed to both evolve together and satisfy the infinite sites assumption.

**Definition 1**. *A rooted tree* T=(V,E) *with vertices* *V* = [*n*] *is an n-clonal tree if each edge* (*i*, *j*) *is labeled by mutation j. We simply write clonal tree when n is clear by context*.

The root r(T) of an *n*-clonal tree T is assigned the unique mutation that does not appear as a label on any edge and is denoted *r* when the tree is clear by context. The vertices and edges of T are denoted as V(T) and E(T). The parent of a non-root, vertex *i* in V(T) is written as *δ*(*i*) and the set of children of a vertex *i* is written as *C*(*i*). The depth *d*(*i*) of a vertex *i* in V(T) is the number of edges on the path from r(T) to *i*. The depth of the tree T is the maximum depth of any vertex *i* in V(T). The clone *b*_*i*_ corresponds to vertex *i* of the *n*-clonal tree and contains all mutations occurring on the unique path from *r* to *i* in T. We summarize all clones in T with an *n-clonal matrix* where each clone is a row of the matrix.

**Definition 2**. *The n-clonal matrix* $\;B_{T}=\left[b_{i,j}\right]$ *is the n-by-n binary matrix such that b*_*i*,*j*_ = 1 *if and only if either* *i* *is a descendant of j in* T *or* *i* = *j*. *We drop the subscript* T *when it is clear by context*.

The model of bulk DNA sequencing is then as follows: each of *m* samples consists of a mixture of distinct clones and the sequencing experiment measures the frequency of all mutations in this mixture. More formally, it is assumed that each of the *m* measurements $f_{i}\in R^{n}$ are *convex combinations* [31] of the *n* clones in T. That is,

$$\begin{array}{c} f_{i}=u_{i,1}b_{1}+u_{i,2}b_{2}+\cdots +u_{i,n}b_{n}\;\text{where}\;u_{i,j}\geq 0\;\text{and}\;\sum_{j=1}^{n}u_{i,j}\leq 1, \end{array}$$ (1)

where clone *b*_*i*_ is the *i*^th^ row of *B*. This model is often summarized compactly in matrix notation as *F* = *UB*, where *F* = [*f*_*i*_] and *U* = [*u*_*i*_] are *m*-by-*n* matrices, *f*_*i*,*j*_ is the frequency of mutation *j* in sample *i*, and *u*_*i*,*j*_ is the fraction of clone *j* in sample *i*. As such, we call *F* a *frequency matrix* and any right stochastic matrix *U* a *usage matrix*.

Under this model, the problem of reconstructing the evolutionary history of the tumor becomes equivalent to factoring the observed frequency matrix *F* into its constituents *U* and *B*. While it would be desirable to solve this factorization problem exactly, imperfect measurement makes this challenging in practice and instead, most methods attempt to infer *U* and *B* such that *F* ≈ *UB*. For example, CITUP [7] attempts to find *U* and *B* such that $\sum_{i=1}^{m}{\left\|F_{i}-{\left(UB\right)}_{i}\right\|}_{2}^{2}$ is minimized. In the general setting, we have a loss function *L*(*F*, *U*, *B*) that provides a measure of error between the observed frequency matrix *F* and the inferred matrices *U* and *B*.

**Problem 1** (The Variant Allele Frequency *L*-Factorization Problem (*L*-VAFFP)). *Given a frequency matrix F, find a clonal matrix B and a usage matrix U such that the loss L*(*F*, *U*, *B*) *is minimized*.

Multiple variations of the *L*-VAFFP have been studied in the literature, with different choices of loss function *L*(*F*, *U*, *B*) and additional constraints, as summarized in Table 1. In particular, CITUP [7] jointly clusters mutations and infers a phylogeny, using a regularized *L*_2_ loss to avoid overfitting on the number of mutation clusters. CITUP is an exact algorithm to solve this problem based off exhaustive enumeration of clonal trees and quadratic integer programming. LICHeE [6] also clusters mutations, and minimizes the total squared violation of the *sum condition* [5–7, 24] in their inferred tree T using quadratic programming. AncesTree [5] on the other hand, does not cluster mutations, and studies two different loss functions. The first loss they study is the 0–1 loss L(F,U,B)=1(F≠UB), which is zero if and only if *F* = *UB*. Interestingly, they show that under this loss function, the variant allele frequency factorization problem is NP-complete, which implies that it is also NP-complete under any *L*_*p*_ loss. As real data is quite noisy, they also study a variant of the *L*_1_ loss obtained from adding the hard constraint that |*F*_*ij*_ − (*UB*)_*ij*_| ≤ *ϵ*_*i*,*j*_ for some *ϵ* > 0. For both loss functions, they use an integer linear programming formulation to solve the problem exactly. CALDER [10] builds off of the approach of AncesTree, but adds an additional hard constraint that the inferred matrices *U* and *B* are *longitudinally consistent*, leveraging the temporal information present in certain experimental settings. Further, rather than minimizing the *L*_1_ loss, they minimize the *L*_0_ loss on the usage matrix *U*. Again, they utilize integer linear programming to solve this optimization problem exactly. A complete description of all methods is summarized compactly in Table 1.

**Table 1:** Summary of several variants of the variant allele frequency factorization problem studied in the literature. The loss functions *L*(*F*, *U*, *B*) and additional constraints beyond U≥0,U1≤1 are noted for each method. Due to space constraints, we do not describe the regularization term(s) appearing in the loss function for several methods [6–9, 11], which penalize the total number of mutation clusters. *CALDER enforces an additional hard constraint that the inferred matrices are *longitudinally consistent*, when provided with additional longitudinal information.

Method	Loss Function *L*(*F*, *U*, *B*)	Additional Constraints	Clusters Mutations?
*fastBE*	$\sum_{i=1}^{m}\;{\left\|F_{i}-{\left(UB\right)}_{i}\right\|}_{1}$	None	No
CITUP [7]	$\sum_{i=1}^{m}{\left\|F_{i}-{\left(UB\right)}_{i}\right\|}_{2}^{2}$	U1=1	Yes
LICHeE [6]	$\sum_{i=1}^{m}\sum_{j=1}^{n}{\left[\text{max}\;\left\{0,\sum_{k\in C(j)}F_{i,k}-F_{i,j}\right\}\right]}^{2}$	None	Yes
AncesTree [5]	1(F≠UB)	None	No
	$\sum_{i=1}^{m}\;{\left\|F_{i}-{\left(UB\right)}_{i}\right\|}_{1}$	|*F*_*ij*_ − (*UB*)_*ij*_| ≤ *ϵ*_*i*,*j*_	No
CALDER*[10]	‖*U*‖_0_	|*F*_*ij*_ − (*UB*)_*ij*_| ≤ *ϵ*_*i*,*j*_	No
	None	|*F*_*ij*_ − (*UB*)_*ij*_| ≤ *ϵ*_*i*,*j*_	No
PhyloWGS [8]	$P_{\text{PhyloWGS}}\left(U,B|F\right)$	None	Yes
PASTRI [9]	$P_{\text{PASTRI}}\left(U,B|F\right)$	None	Yes
Pairtree [11]	$P_{\text{Pairtree}}\left(U,B|F\right)$	None	Yes
Orchard [12]	$P_{\text{Orchard}}\left(U,B|F\right)$	None	No

The main focus of this work is the *structured regression* problem that appears when the clonal matrix *B* is fixed. Specifically, when *B* is fixed, the problem reduces to finding a usage matrix *U* such that the loss *L*(*F*, *U*, *B*) is minimized. We call this a *structured regression* problem as the aim is to *regress* the frequency matrix *F* against the clonal matrix *B* where *B* has a unique, *combinatorial structure* which we describe in Section 2.2.1.

**Problem 2** (The Variant Allele Frequency *L*-Regression Problem (*L*-VAFRP)). *Given a frequency matrix F and a clonal matrix B, find a usage matrix U such that the loss L*(*F*, *U*, *B*) is minimized. We call this minimum loss L*(*F*, *B*).

In the special case where the loss $L_{p}\left(F,U,B\right)≔\sum_{i=1}^{m}\;{\left\|F_{i}-{\left(UB\right)}_{i}\right\|}_{p}^{p}$ for *p* ∈ {1, 2}, this regression problem is solveable in polynomial time. More formally, since *u*_*i*,*j*_ ≥ 0 and $\sum_{j=1}^{n}\;u_{i,j}\leq 1$ are linear constraints (1) on the matrix *U*, the *L*_1_ and *L*_2_ regression problems can be formulated as linear and convex quadratic programs respectively, which are both solveable in polynomial time. Throughout the remainder of this work, we will focus on the *L*_1_ regression and factorization problems, which we call the *variant allele frequency ℓ*_1_-*regression problem* (*ℓ*_1_-VAFRP) and *variant allele frequency ℓ*_1_-*factorization problem* (*ℓ*_1_-VAFFP), respectively. Further, though a slight abuse of notation, we will write ${\left\|F-UB\right\|}_{p}^{p}$ instead of *L*_*p*_(*F*, *U*, *B*).

In contrast to the aforementioned approaches, which preprocess the read count data to obtain the observed frequency matrices *F*, probabilistic approaches such as PhyloWGS [8], PASTRI [9], Pairtree [11], and Orchard [12] explicitly model the read count data. Further, rather than minimize a loss, these methods attempt to sample from the posterior distribution of phylogenetic trees using a variety of sampling techniques. Since these probabilistic approaches are challenging to succinctly describe, we refer to the original publications [8, 9, 11, 12] for a complete description and denote their loss generically as P(U,B∣F) in Table 1.

General purpose linear programming software solves the *ℓ*_1_-VAFRP in polynomial time. However, linear programming solvers do not exploit the special structure of *B* and have worse asymptotic complexity as compared to the algorithm we derive in this work. Numerical methods from convex optimization such as the Alternating Direction Method of Multipliers and Projected Gradient Descent Method [31] can be used to solve the *L*_2_ variant allele frequency regression problem to a guaranteed optimality threshold. However, these blackbox methods are again quite slow and do not yield an exact solution. As such, [32] designed an improved algorithm that solves the *L*_2_ regression problem exactly in $O(mn^{2})$ time.

### 2.2 A structured regression model for the *ℓ*_1_-VAFFP

**Fig 1:**
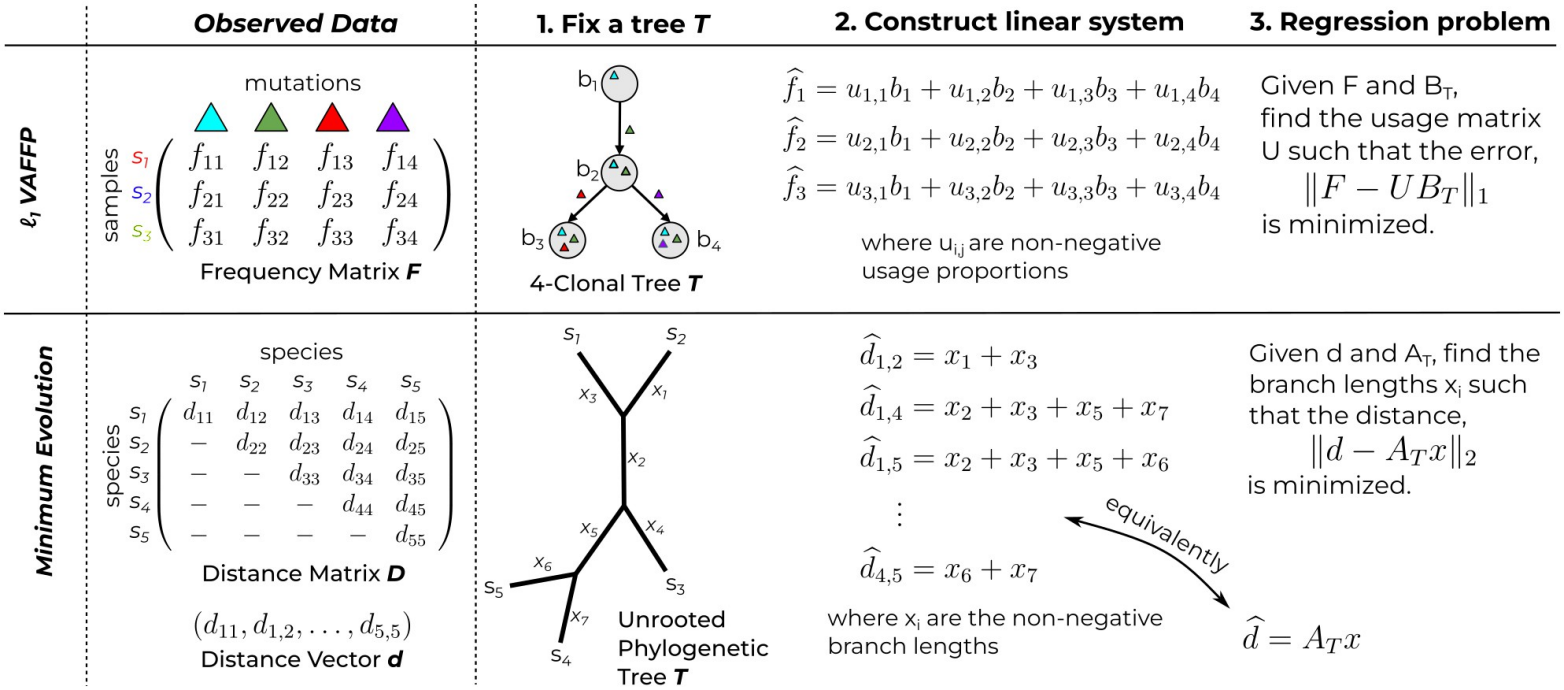
Analogous *structured regression* problems for a fixed tree topology *T* in the: (top) *ℓ*_1_-VAFFP framework for cancer evolution and the (bottom) minimum evolution framework for distance based phylogenetics. The usage matrix *U* describes the fraction of each clone across all samples, and the clonal matrix *B*_*T*_ describes the genotype of each clone in *T*. The matrix *A*_*T*_ describes the system of linear equations relating the branch lengths *x* to the distance vector *d*.

Structured regression and local search have played a pivotal role in the success of methods for performing distance based reconstruction of phylogenetic trees. In particular, state-of-the-art methods for distance based phylogenetics such as FastME [33] and FastTree [34, 35] work by locally exploring phylogenetic tree space (i.e. a tree space polytope [36, 37]) and regressing the observed distances against the tree to infer the unknown branch lengths, picking the tree which best explains the observed distances (Fig 1). Pivotal to the success of these methods are efficient algorithms [20, 38, 39] for computing and recomputing the solution to the structured regression problem. Importantly, these algorithms leverage the structured relationship between the branch lengths, the tree, and the induced distances.

Despite the success of structured regression as a tool for distance based phylogenetics, such an approach has not yet been studied in the context of tumor evolution. Here, we derive an efficient algorithm for the *ℓ*_1_-VAFRP by exploiting the combinatorial structure of clonal matrices. In particular, we start by studying the structure of clonal matrices, both summarizing and extending existing results. Then, narrowing our focus, we derive an equivalent characterization of the *ℓ*_1_-VAFRP, which emphasizes the tree structure inherent in the problem. Finally, we use our characterization to design an efficient algorithm for the *ℓ*_1_-VAFRP which enables fast recomputation upon subtree prune-and-regraft (SPR) operations [23], providing the main theoretical result of our work, as stated below.

**Theorem 1**. *Given a clonal tree* T *with n vertices and an m-by-n frequency matrix F, the minimum*

$$\begin{array}{c} L_{1}^{*}\left(F,B_{T}\right)=\text{min}\;\{{\left\|F-UB_{T}\right\|}_{1}:U\geq 0,U1\leq 1\} \end{array}$$ (2)

*can be found in* O(mnd) *time, where d is the depth of* T.

As mentioned above, our algorithm for solving the *ℓ*_1_-VAFRP is also able to efficiently recompute the minimum $L_{1}^{*}\left(F,B_{T}\right)$ upon slight modifications to the tree topology, in the sense of the following corollary.

**Corollary 1**. *Given a clonal tree* T *with* *n* *vertices and an m-by-n frequency matrix F, the following queries can be efficiently answered after* O(mnd) *pre-processing time using* O(mnd) *space*.

*(i) For a subtree prune-and-regraft (SPR) operation on vertices i and j which results in a tree* $T^{\prime }$ , *the minimum* $L_{1}^{*}\left(F,B_{T^{\prime }}\right)$ *can be queried in* O(md·max{d(i),d(j)}) *time*.
*(ii) For the operation of attaching a new vertex j as a child of a vertex i to obtain a tree* $\;T^{\prime }$ *and appending a corresponding column to the frequency matrix F to obtain F*′, *the minimum* $L_{1}^{*}\left(F^{\prime },B_{T^{\prime }}\right)$ *can be queried in* O(md·d(i)) *time*.

Importantly, the depth *d* of a tree on *n* vertices is at most *n* − 1, implying our algorithm runs in quadratic (in *n*) time in the worst case and improves upon linear programming based approaches. However, for reasonable classes of trees, the complexity is much better. For example, the expected depth of a rooted spanning tree drawn uniformly at random from the complete graph *K*_*n*_ is of order $\sqrt{n}\;\text{log}\;n$—similar bounds can also be derived for the random spanning trees of an arbitrary graph *G* [40, 41]. All proofs can be found in Supplementary Results A in S1 Text.

#### 2.2.1 Clonal trees and matrices

The most salient feature of clonal matrices is that they are (two-state) perfect phylogeny matrices [5], and this allows us to tap into the theory which studies such matrices. However, the class of clonal matrices is much more restrictive: there are a handful of useful results concerning clonal matrices which are not applicable to perfect phylogeny matrices. For example, a perfect phylogeny matrix need not be square and thus it is not necessarily invertible, while clonal matrices are always invertible [5].

We continue the study of clonal matrices by drawing an analogy to perfect phylogeny. Specifically, we introduce a new, recursive definition of clonal matrices that is inspired by the recursive definition of perfect phylogeny matrices described by Gusfield [25, 26]. Formally, we show that a matrix *B* is an *n*-clonal matrix if and only if the rows and columns of *B* can be reordered such that *B* is *clonally canonical*, a term we define below.

**Definition 3**. *A matrix* *B* *is clonally canonical if i)* *B*_*i*,1_ = 1 *for all i* ∈ [*n*], *ii) B*_1,*j*_ = 0 *for all j* ∈ {2, …, *n*}, *and iii) there exists clonally canonical matrices* *B*_1_, *B*_2_, …, *B*_*k*_ *such that B*_2:*n*,2:*n*_ *is block diagonal with blocks* *B*_1_, *B*_2_, …, *B*_*k*_. *That is, B is clonally canonical if it has the form*:

$$B=(\begin{array}{cc} \begin{array}{c} 1 \end{array} & \begin{array}{ccc} 0 & \cdots & 0 \end{array}\\ \begin{array}{c} 1\\ \vdots \\ 1 \end{array} & \begin{array}{ccc} B_{1} & & 0\\ & \ddots & \\ 0 & & B_{k} \end{array} \end{array})$$

Conveniently, for a given clonal tree T, it is straightforward to construct a clonally canonical matrix associated with T by relabeling the clones by their preorder traversal index during a depth first search starting at *r*. With this definition, we are now ready to state several equivalent characterizations of clonal matrices.

**Proposition 1**. *For any binary n-by-n matrix B, the following conditions are equivalent*:

*(i) B is an n-clonal matrix*.
*(ii) The rows and columns of B can be reordered to make B clonally canonical*.
*(iii) B satisfies the following conditions* [5]:
  *(a) There exists exactly one index r such that* $\sum_{j=1}^{n}B_{r,j}=1$.
  *(b) For all i* ≠ *r, there exists exactly one j such that B*_*j*,*k*_ = 1 *implies B*_*i*,*k*_ = 1 *and* $\sum_{k=1}^{n}(B_{i,k}-B_{j,k})=1$.
  *(c) B*_*i*,*i*_ = 1 *for all indices i* ∈ [*n*].

The clonally canonical form of the clonal matrix associated with a clonal tree T is especially useful since it enables efficient multiplication and inversion of *B*.

**Proposition 2**. *Let B be an n-by-n clonally canonical matrix associated with the clonal tree* T. *Then, the four matrix vector products*

$$Bv,\;v^{T}B,\;B^{-1}v\;and\;v^{T}B^{-1}$$

*can be computed in* O(n) *time for any vector* $v\in R^{n}$ *when given the clonal tree* T.

We conclude this section with an algebraic description of the inverse of *B*, which was previously described in [32], and will serve as a key technical ingredient for deriving an equivalent formulation of the *ℓ*_1_-VAFRP. Define the adjacency matrix *A* of T as the *n*-by-*n* matrix such that *A*_*i*,*j*_ = 1 if *i* is a parent of *j* in T and 0 otherwise. Then, the inverse of *B* is (*I* − *A*), where *I* is the identity matrix.

**Lemma 1**. *For any clonal matrix B associated with a clonal tree* T *having adjacency matrix A*, *B* = (*I* − *A*)^−1^. *Consequently*, [*B*^−1^*v*]_*i*_ = *v*_*i*_ − *v*_*δ*(*i*)_ *if* i≠r(T), *and* [*B*^−1^*v*]_*i*_ = *v*_*i*_ *when* i=r(T).

#### 2.2.2 An equivalent formulation of the VAF *ℓ*_1_-regression problem

In this section, we show that the *ℓ*_1_-VAFRP is equivalent to a constrained vertex labeling problem on the clonal tree T associated with *B*. Specifically, we prove that the *ℓ*_1_-VAFRP is equivalent to a special case of the *dot product tree labeling problem*, defined as follows, for the case when *m* = 1 and *F* = *f*^*T*^. The general case of *m* > 1 follows from the separability of the objective $\sum_{i=1}^{m}\;{\left\|F_{i}-{\left(UB\right)}_{i}\right\|}_{1}$.

**Problem 3** (Dot Product Tree Labeling Problem (DPTLP)). *Given a rooted tree* T *with vertices* [*n*] *and a vector* $w\in R^{n}$, *find a non-negative vector* $x\in R^{n}$ *such that* *x*^*T*^*w* *is maximized and* |*x*_*i*_ − *x*_*δ*(*i*)_| ≤ 1 *for all* i≠r(T), *where* *δ*(*i*) *is the parent of vertex i in* T.

**Fig 2:**
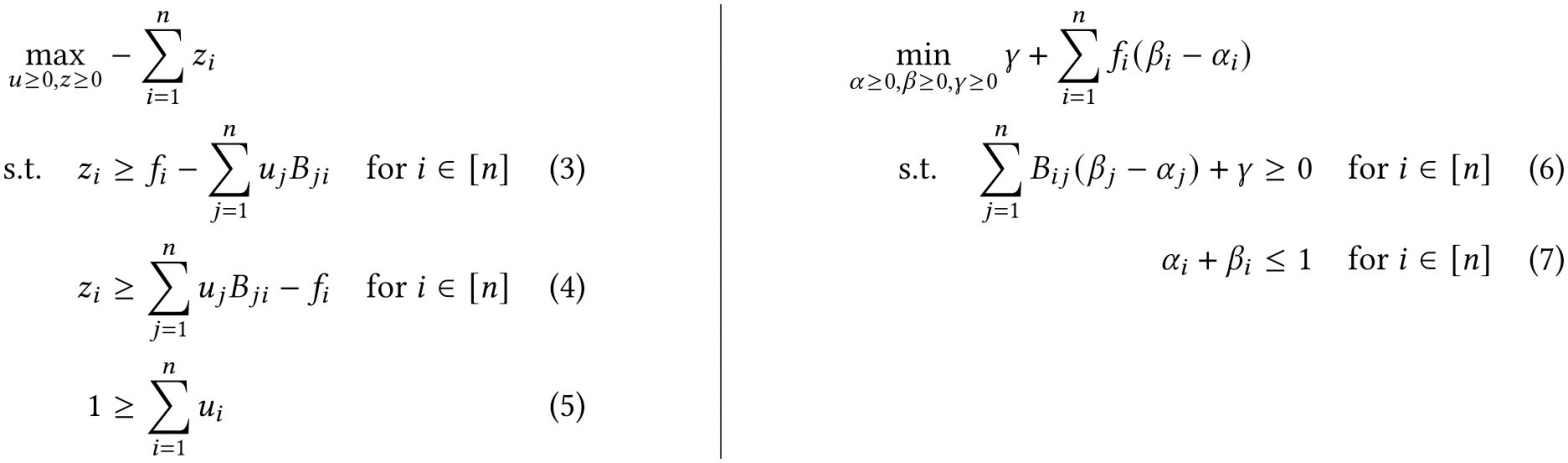
(Left) The primal LP and (right) the dual LP for the *ℓ*_1_-VAFRP.

To derive the equivalence between the *ℓ*_1_-VAFRP and the DPTLP, we start by writing the *ℓ*_1_-VAFRP as a linear program (LP) in standard form [31]. Using the usual trick [31] for converting the *ℓ*_1_ norm to a linear objective with linear constraints, we write the *ℓ*_1_-VAFRP as a LP (Fig 2). Then, we write out the dual problem by associating a dual variable *α*_*i*_ with the constraint in (3), a dual variable *β*_*i*_ with the constraint in (4), and a dual variable *γ* with the constraint in (5). Thus obtaining our dual LP (Fig 2).

To simplify the dual form of the LP, we perform a change of variables by setting λ_*i*_ = *β*_*i*_ − *α*_*i*_. Since *α*_*i*_ and *β*_*i*_ are non-negative and their sum is bounded by 1, λ_*i*_ ∈ [−1, 1]. Then, writing the constraints in matrix form and using a slack variable *ψ* to remove the inequality constraint, we have the following equivalent, dual LP.

$$\begin{array}{cc} \underset{\gamma \geq 0,\psi \geq 0}{\text{min}}\;\gamma +f^{T}\lambda \\ \text{subject}\;\text{to}\;B\lambda & =\psi -\gamma 1 \end{array}$$ (8)

$$\begin{array}{c} \lambda_{i}\in [-1,1]\;\text{for}\;i\in [n] \end{array}$$ (9)

Applying Lemma 1 to the matrix *B* in (8) and invoking LP duality then proves the following theorem.

**Theorem 2**. *Given a length n frequency vector f and an n-by-n clonal matrix B corresponding to a clonal tree with root vertex* 1, *the minimum of* ‖*f*^*T*^ − *u*^*T*^*B*‖_1_ *over all usage vectors* $u\in R^{n}$ *is equal to the maximum of*

$$\begin{array}{c} w^{T}x\;s.t.\;w_{i}=\{\begin{array}{cc} -[f_{i}-\sum_{j\in C(i)}f_{j}] & if\;i\neq n+1,\\ f_{1}-1 & otherwise. \end{array} \end{array}$$ (10)

*over all non-negative vectors* $x\in R^{n+1}$ *such that* |*x*_1_ − *x*_*n*+1_| ≤ 1 *and* |*x*_*i*_ − *x*_*δ*(*i*)_| ≤ 1 *for all* *i* ∈ [*n*].

In other words, the above theorem states that the *ℓ*_1_-VAFRP is equivalent to a special case of the DPTLP where we append a parent labeled *n* + 1 to the root vertex and appropriately set the vector $w\in R^{n+1}$. Interestingly, the *sum condition* [5–7, 24], that is, the requirement that

$$\begin{array}{c} f_{i}\geq \sum_{j\in C(i)}f_{j}, \end{array}$$ (11)

appears almost unexpectedly in (10). Using the appearance of the sum condition in (10), we extend the theory of El-Kebir et al. [5] which states that we can find a usage vector *u*^*T*^ such that *f*^*T*^ = *u*^*T*^*B* if and only if the sum condition (11) is satisfied. In particular, we show that the total violation of the sum condition also provides a lower bound on the *ℓ*_1_ error.

**Corollary 2**. *Let f be a frequency vector of length n, let B be an n-by-n clonal matrix, and let* $L_{1}^{*}\left(f^{T},B\right)=\text{min}\;\left\{{\left\|f^{T}-u^{T}B\right\|}_{1}:u\geq 0,u^{T}1\leq 1\right\}$. *Then* $L_{1}^{*}\left(f^{T},B\right)\geq \sum_{i=1}^{n}\;\text{max}\;\{\sum_{j\in C(i)}f_{j}-f_{i},0\},$ *or the total violation of the sum condition* (11). *Thus*, $L_{1}^{*}\left(f^{T},B\right)=0$ *if and only if the sum condition is satisfied*.

Finally, we observe that as a consequence of the above corollary, the sum condition is somewhat redundant when minimizing the *ℓ*_1_ error ‖*F* − *UB*‖_1_. This is because by the above corollary, the *ℓ*_1_ error is an upper bound on the total violation on the sum condition, implying that minimizing the *ℓ*_1_ error forces the total violation of the sum condition to zero.

#### 2.2.3 An algorithm for the DPTLP

In this section, we develop an efficient algorithm for the DPTLP. There are two key ideas underlying our algorithm. The first idea is that exploiting the tree structure enables us to express the solution for the subtree rooted at a vertex *i* in terms of the solution for the subtrees rooted at the children of *i*. This expression is derived using standard techniques for dynamic programming on trees [42]. The second idea is more technical, and is based on the observation that the solution of this recurrence is a concave piecewise linear function, which we represent compactly as a list of size O(d), where *d* is the depth of T. Combining these two ideas yield an O(nd) algorithm for the DPTLP, as stated below.

**Theorem 3**. *Given a rooted tree* T *of depth d with vertices* [*n*] *and a vector* $w\in R^{n}$, *the DPTLP can be solved in* O(nd) *time with the following recurrence*,

$$\begin{array}{c} g_{i}\left(\psi \right)=\psi \cdot w_{i}+\sum_{j\in C(i)}\underset{\left|\psi_{j}-\psi \right|\leq 1,\psi_{j}\geq 0}{\text{max}}\;g_{j}\left(\psi_{j}\right), \end{array}$$ (12)

*where g*_*i*_(*ψ*) *is the optimal solution to the DPTLP for the subtree rooted at vertex i, when vertex i is assigned the label ψ*.

We will prove this theorem by first deriving a recurrence relation for the solution of the DPTLP. Assume *g*_*i*_(*ψ*) is the optimal solution to the DPTLP for the subtree rooted at *i* when the root vertex *i* is assigned the label *ψ*. Then, *g*_*i*_(*ψ*) satisfies the following recurrence relation:

$$\begin{array}{cccc} g_{i}\left(\psi \right) & =\psi \cdot w_{i}+\underset{\left|\psi_{j}-\psi \right|\leq 1,\psi_{j}\geq 0}{\text{max}}\;\{\sum_{j\in C(i)}g_{j}\left(\psi_{j}\right)\} & & \text{by}\;\text{definition,}\\ & =\psi \cdot w_{i}+\sum_{j\in C(i)}\;\underset{\left|\psi_{j}-\psi \right|\leq 1,\psi_{j}\geq 0}{\text{max}}\;g_{j}\left(\psi_{j}\right) & & \text{by}\;\text{separability},\\ & =\psi \cdot w_{i}+\sum_{j\in C(i)}h_{j}\left(\psi \right), & & \text{by}\;\text{defining}\;h_{j}\left(\psi \right)≔\underset{\left|\psi_{j}-\psi \right|\leq 1,\psi_{j}\geq 0}{\text{max}}\;g_{j}\left(\psi_{j}\right). \end{array}$$ (13)

Importantly, to solve the DPTLP, it is necessary and sufficient to compute max_*ψ*≥0_*g*_*r*_(*ψ*) for the root vertex r=r(T). Unfortunately, however, the straightforward technique [42] of storing a dynamic programming table for this recurrence will not work because the number of possible values of *ψ* ≥ 0 is infinite. Thus, we need to find an alternative way to describe and represent the functions *g*_*j*_ and *h*_*j*_ appearing in this recurrence.

The key mathematical idea underpinning our algorithm is that the functions *g*_*j*_ and *h*_*j*_ appearing in the recurrence are not arbitrary. Rather, they form a special class of functions which admit a compact representation and are convenient to work with. In particular, the functions *g*_*j*_ and *h*_*j*_ are concave (and thus continuous) piecewise linear functions with a finite number of breakpoints at coordinates {1, 2, …, *k*}, formally defined below.

**Definition 4**. *Let* $L_{k}$ *be the set of concave piecewise linear functions with breakpoints at integers* 1, …, *k; i.e., a continuous function* $f\in L_{k}$ *if f is linear with slopes s*_1_ ≥ *s*_2_ ≥ … ≥ *s*_*k*+1_ *on the intervals I*_1_ = [0, 1), *I*_2_ = [1, 2), …, *I*_*k*+1_ = [*k*, ∞) *respectively*.

Note that $L_{k}$ form a strictly increasing nested sequence $L_{0}\subset L_{1}\subset \ldots$ of sets of piecewise linear functions. Further, every function *f* in the class $L_{k}$ is represented by a tuple (*y*, *s*_1_, …, *s*_*k*+1_) of size *k* + 2 giving the intercept *y* and slopes *s*_*i*_ of each piece of *f*.

Now, we will prove that *g*_*j*_ and *h*_*j*_ are in the class $L_{d}$, where *d* is the depth of T. To start, notice that if *i* is a leaf vertex, then *g*_*i*_(*ψ*) = *ψ* ⋅ *w*_*i*_ is a linear function and is thus in $L_{0}$. This provides us with the necessary base case required to prove that *g*_*j*_ is in $L_{d}$. Next, we prove the inductive step of our claim: if $g_{j}\in L_{k}$, then $h_{j}\in L_{k+1}$. The results then follow by induction on the depth of T. We start with the following description of *h*_*j*_ in terms of *g*_*j*_.

**Lemma 2**. *Suppose f* ∈ *L*_*k*_ *and is represented by the tuple* (*y*, *s*_1_, *s*_2_, …, *s*_*k*+1_). Let i* *be the largest index i such that s*_*i*_ ≥ 0. *If s*_*i*_ < 0 for all i, we set i* = −∞ *and if s*_*i*_ ≥ 0 for all i, we set i* = ∞. *Then*,

$$h\left(\psi \right)≔\underset{\left|\psi^{\prime }-\psi \right|\leq 1,\psi^{\prime }\geq 0}{\text{max}}\;f\left(\psi^{\prime }\right)$$

*is in* $L_{k+1}$ *and satisfies*

$$\begin{array}{c} h\left(\psi \right)=\{\begin{array}{cc} \;f(i^{*}) & \text{if}\;\psi \in [i^{*}-1,i^{*}+1],\\ \;f(\psi +1) & \text{if}\;\psi <i^{*}-1,\\ \;f(\text{max}\;\{\psi -1,0\}) & \text{if}\;\psi >i^{*}+1. \end{array} \end{array}$$ (14)

*Proof*. If i* = ∞, all slopes *s*_*i*_ are non-negative and the function *g*(*ψ*′) is non-decreasing. Thus, the maximum of *g*(*ψ*′) over any interval [*ψ* − 1, *ψ* + 1], is achieved at the interval’s right most value *ψ* + 1.

If i* = −∞, all slopes *s*_*i*_ are negative and the function *g*(*ψ*′) is strictly decreasing. Thus, the maximum of *g*(*ψ*′) over any interval [*ψ* − 1, *ψ* + 1], is achieved at the interval’s left most value *ψ* − 1. However, since *ψ*′ is constrained to be non-negative, if *ψ* < 1 the maximum is achieved at *ψ*′ = 0.

If i* ≠ ∞, −∞, then *g*(*ψ*′) is non-decreasing on the interval [0, i*] and non-increasing on the interval [i*, ∞). Further, the maximum of *g*(*ψ*′) over all non-negative *ψ*′ is bounded and equal to *g*(i*). The result then follows by a case analysis on the value of *ψ*. If *ψ* is in [i* − 1, i* + 1], then we can take *ψ*′ = i* and achieve the maximum. If *ψ* < i* − 1, then the function *g*(*ψ*′) is non-decreasing on the interval [*ψ* − 1, *ψ* + 1] and the maximum is achieved at the interval’s right most value *ψ* + 1. Symetrically, if *ψ* > i* + 1, then the function *g*(*ψ*′) is non-increasing on the interval [*ψ* − 1, *ψ* + 1] and the maximum is achieved at the interval’s left most value *ψ* − 1, which is always non-negative since *ψ* > 1. As this covers all possible cases, this proves that *h* has the form (14).

To see that *h*(*ψ*) is continuous, observe that

$$\underset{\psi \to {\left(i^{*}-1\right)}^{-}}{\text{lim}}\;h\left(\psi \right)=g\left(i^{*}\right)\;\text{and}\;\underset{\psi \to {\left(i^{*}+1\right)}^{+}}{\text{lim}}\;h\left(\psi \right)=g\left(i^{*}\right)$$

at the only candidates for discontinuity, i* − 1 and i* + 1. To see that it is concave, note that

$$h^{\prime \prime }\left(\psi \right)=\{\begin{array}{cc} \;0 & \text{if}\;\psi \in [i^{*}-1,i^{*}+1],\\ \;g^{\prime \prime }\left(\psi +1\right) & \text{if}\;\psi <i^{*}-1,\\ \;g^{\prime \prime }\left(\psi -1\right) & \text{if}\;\psi >i^{*}+1. \end{array}$$

As *g*(*ψ*) is concave, its second derivative *g*″(*ψ*) is non-positive, which implies that *h*(*ψ*) is also concave. Since *h* is trivially piecewise linear by (14), the proof is complete.

Stated in terms of the tuple representations of *h*_*j*_ and *g*_*j*_ as tuples, we have the following equivalent result.

**Proposition 3**. *Suppose f* ∈ *L*_*k*_ *and is represented by the tuple* (*y*, *s*_1_, *s*_2_, …, *s*_*k*+1_). Let i* *be the largest index i such that s*_*i*_ ≥ 0. *If* *s*_*i*_ < 0 for all i, we set i* = −∞ *and if s*_*i*_ ≥ 0 for all i, we set i* = ∞. *Then*,

$$h\left(\psi \right)≔\underset{\left|\psi^{\prime }-\psi \right|\leq 1,\psi^{\prime }\geq 0}{\text{max}}\;f\left(\psi^{\prime }\right)$$

*is in* $L_{k+1}$ *and is represented by the tuple*

$$\begin{array}{cc} (y+s_{1},s_{2},s_{3},\ldots ,s_{k})\; & if\;i^{*}=\infty ,\\ (y,0,s_{1},s_{2},s_{3},\ldots ,s_{k})\; & if\;i^{*}=-\infty ,\\ (y+s_{1},s_{2},s_{3},\ldots ,s_{i^{*}-1},0,0,s_{i^{*}+1},s_{i^{*}+2},\ldots ,s_{k})\; & otherwise. \end{array}$$

In summary, we have shown that *i)* if *i* is a leaf, *g*_*i*_ is in $L_{0}$, and *ii)* if *g*_*j*_ is in $L_{k}$, then *h*_*j*_ is in $L_{k+1}$. The final step is to show that if the function *h*_*j*_ is in $L_{k}$ for all children *j* of *i*, then *g*_*i*_ is also in $L_{k}$. This follows from the observation that the class $L_{k}$ is closed under addition. The result is summarized below.

**Proposition 4**. *Let g*_*i*_(*ψ*) *be the optimal solution of the DPTLP for the subtree* $T_{i}$ *rooted at vertex i such that i is assigned the label ψ*. *Then, g*_*i*_ *is in* $L_{d}$.

We are now ready to prove the main result of this section.

*Proof of Theorem 3*. The result follows by induction on *n*, the number of vertices in T. In particular, assume that the representation of the function *g*_*i*_ for the root vertex *i* is computable in O(nd) time for all trees with fewer than *n* vertices and depth at most *d*. Clearly, this holds for *n* = 1.

Then, let *r* be the root of T and let *C*(*r*) be the set of children of *r*. Let $T_{j}$ denote the subtree rooted at *j* ∈ *C*(*r*) and *n*_*j*_ denote the size of $T_{j}$. By the inductive hypothesis, we can compute the representation of *g*_*j*_ for *j* ∈ *C*(*r*) in $O(dn_{j})$ time, since the depth of the subtree rooted at *j* ∈ *C*(*r*) is at most *d* + 2. Using Proposition 3, we can compute the representation of *h*_*j*_ for all *j* ∈ *C*(*r*) in $O\left(d\sum_{j\in C(r)}n_{j}\right)=O\left(nd\right)$ time as these functions are represented by tuples of length at most *d* + 3.

Finally, we compute the representation of *g*_*r*_ by observing that $L_{d}$ is closed under addition and that the representation of *g*_*r*_ is easily computed from the representations of *h*_*j*_. In particular, summing the tuples representing *h*_*j*_ coordinate-wise, we obtain the representation of *g*_*r*_.

Then, by reducing the *ℓ*_1_-VAFRP to the DPTLP using Theorem 2 and applying Theorem 3, we complete the proof of the first part of Theorem 1 for the special case where *m* = 1. The general case of *m* > 1 follows by noting that the objective $\sum_{i=1}^{m}\;{\left\|F_{i}-{\left(UB_{T}\right)}_{i}\right\|}_{1}$ is separable and that each term ${\left\|F_{i}-{\left(UB_{T}\right)}_{i}\right\|}_{1}$ can be minimized independently.

To see Corollary 1, notice that after pruning and regrafting any subtree $T_{i}$ as a child of a vertex *j*, *g*_*k*_ only changes if the vertex *k* is on the path from root r(T) to the vertex *δ*(*i*) or the vertex *j*—this follows from Eq (13). Since there are at most 2 ⋅ max{*d*(*j*), *d*(*i*)} vertices on these paths, this observation yields the statement (i) in Corollary 1, as long as the representations of *g*_*k*_ are stored. The second statement (ii) in Corollary 1 follows by a similar argument.

### 2.3 A deterministic search algorithm for the *ℓ*_1_-VAFFP

Here we describe a deterministic search algorithm, *fastBE*, for the *ℓ*_1_-VAFFP which builds upon the fast regression algorithm described in Section 2.2 and is inspired by the beam search techniques used by Orchard [12]. Our algorithm iteratively constructs the inferred tree one mutation at a time, choosing the best vertex placement for each mutation in the current tree, while allowing added vertices to “adopt” children from their parent. A vertex *u* is said to *adopt* the child *w* of a vertex *v* in T upon removing edge (*v*, *w*) and adding the edge (*u*, *w*) to T. This procedure is described formally as follows:

1. Fix an order *O* = {*o*_1_, …, *o*_*n*_} in which to append the mutations *O* and initialize the starting tree as $T_{1}=\left(\left\{o_{1}\right\},\left\{\right\}\right)$ with root $r\left(T_{1}\right)=o_{1}$.
2. For *i* = 2, …, *n*:
  (a) Let the current tree $T=T_{i-1}$ and the current frequency matrix *F*′ be the submatrix of *F* spanned by the columns *o*_1_, …, *o*_*i*_.
  (b) Find the parent p∈V(T) and the subset of children $S\subseteq \delta_{T}\left(p\right)$ such that the tree $T^{\prime }$ obtained from attaching *o*_*i*_ as a child of *p* in T and adopting the children *S* as children of *o*_*i*_ minimizes the loss $L^{*}\left(F^{\prime },B_{T^{\prime }}\right)$.
  (c) Set the next tree $T_{i}=T^{\prime }$.
3. Output the final tree $T_{n}$.

Since at every iteration *i* of the algorithm there are a total of $O(n2^{\Delta^{*}})$ where $\Delta^{*}={\text{max}}_{u\in V(T_{i})}\;\left|\delta (u)\right|$ placements of mutation *o*_*i*_ to consider, the running time of this algorithm is dominated by the time to compute the objective function $L_{1}^{*}\left(F,B\right)$ over all such placements. Using the efficient recomputation procedure outlined in the latter part of Theorem 1, we can avoid the naïve approach which takes $O(mn^{2}2^{\Delta^{*}}d)$ time and instead perform all such computations in $O(mn2^{\Delta^{*}}{\tilde{d}}^{2})$ time, where $\tilde{d}$ is the average depth of the tree $T_{i}$. Further details on these steps are supplied in Section B.4 in S1 Text.

### 2.4 Inference of mutation clusters with phylogeny constrained clustering

The scalability of *fastBE* enables the inference of clone trees directly from mutation-level read count data, foregoing the need to cluster mutations prior to running *fastBE*. Still, it is often desirable to cluster similar mutations together, for example, to improve interpretability or to estimate tumor heterogeneity. Typically, clustering of mutations is done with specialized tools such as PyClone [28, 29], SciClone [30], EXPANDS [43], or QuantumClone [44]. However, these tools do not exploit the phylogenetic relationships between the mutations, as thus far, no method was able to infer clone trees at mutation granularity.

To incorporate phylogenetic information, we formalize the mutation clustering problem in a k-means fashion, with the additional constraint that the selected mutation clusters form *connected components* on the inferred clone tree.

**Problem 4** (*p*-Phylogeny Constrained Mutation Clustering (*p*-PCMC)). *Given an n-clonal tree* T, *an m-by-n frequency matrix F*, *and a number of clusters* k∈N, *find a clustering C* = {*C*_1_, …, *C*_*k*_} *of the vertices* V(T) *and a set of cluster centers* $c_{1},\ldots ,c_{k}\in R^{m}$ *such that the loss*

$$\begin{array}{c} \sum_{l=1}^{k}\sum_{j\in C_{l}}{\left\|F_{:,j}-c_{l}\right\|}_{p}^{p} \end{array}$$ (15)

*is minimized, the clustering C partitions* V(T), *and each cluster C*_*i*_ *forms a connected component in* T.

Due to the constraint that the clusters *C*_*i*_ form connected components in T, we identify each clustering with a set of *k* − 1 edges describing the cut of the partition *C*. Consequently, there are $\left(\begin{array}{c} n\\ k-1 \end{array}\right)$ possible clusterings to the *p*-PCMC problem, corresponding to each selection of *k* − 1 edges. Further, for a fixed clustering *C*, the optimal centers *c*_*i*_ are obtained by taking the median vector if *p* = 1 and the mean vector if *p* = 2. Thus, the *p*-PCMC is solveable in polynomial time for fixed *k* when *p* ∈ {1, 2} by checking all $O(n^{k-1})$ possible clusterings.

While the *p*-PCMC is solveable in polynomial time for fixed *k* where *p* ∈ {1, 2}, this naiv̈e solution is computationally prohibitive for clone trees containing upwards of *n* = 1000 mutations and *k* = 20 clones. Consequently, we use a heuristic approach to solve the *p*-PCMC problem, which exploits the fact that the *p*-PCMC problem is solvable in $O(mn^{2})$ time for *k* = 2. In particular, we use a divisive clustering algorithm [45], which builds a clustering by recursively splitting the clustering until *k* clusters are formed. Divisive clustering runs in the opposite direction to the more frequently used hierarchical, or agglomerative, clustering and is typically not used because finding the optimal split of an existing cluster is a hard problem. However, due to the phylogenetic constraints imposed by the *p*-PCMC, we are able to optimally solve the splitting step in $O(mn^{2})$ time, leading to an extremely effective, $O(mn^{2}k)$ time algorithm for the *p*-PCMC problem which is optimal when *k* = 2.

## 3 Results

### 3.1 Runtime comparison to linear programming solvers

We compared an implementation of our structured regression algorithm for the *ℓ*_1_-VAFRP to a linear programming (LP) approach on simulated data. In particular, we implemented the natural primal LP formulation solving the *ℓ*_1_-VAFRP (Section 2.2.2) using two commercial LP solvers: Gurobi v9.0.3 [46] and CPLEX v22.1.0 [47]. We generated 264 pairs of frequency matrices *F* and clonal matrices *B* as described in Section B.1 in S1 Text, and measured the wall-clock runtime of our algorithm and the LP solvers on these simulated instances. Excluding the time required to construct the LP—which would unfairly penalize the LP solvers—we found that our algorithm was a mean of 95.6 times faster than Gurobi and 105.1 times faster than CPLEX (S1 and S2 Figs).

Next, we tested the warm start capability of our structured regression algorithm for the *ℓ*_1_-VAFRP upon perturbations to the topology of the input tree. For each of the 264 instances constructed above, we measured the time to solve the *ℓ*_1_-VAFRP for 25,000 trees obtained by applying a single random SPR operation to the input clonal tree. We performed this measurement both in the setting where we employ our regression algorithm as a black-box (the *cold start* setting) and the setting where we used the recomputation procedure outlined in Corollary 1 (the *warm start* setting). We found that our algorithm was a mean of 6.2 times faster in the warm start as opposed to cold start settings (S3 and S4 Figs). This implies that our regression algorithm possesses another advantage over naïve LP approaches, which do not provide any warm startingcapabilities.

### 3.2 Evaluation of *fastBE* on simulated data

We evaluated our factorization algorithm, *fastBE*, on simulated data, and compared it to four other state-of-the-art factorization algorithms: Pairtree [11], Orchard [12], CALDER [10], and CITUP [7]. To perform our evaluation, we simulated ground truth clone trees and usage matrices, and measured the ability of each algorithm to reconstruct this ground truth. To construct each simulated instance, we generated a clone tree T and usage matrix *U*, computed the frequency matrix *F* = *UB*, and sampled both variant and non-variant reads from *F* at 40× coverage. Complete details describing the simulations, parameters, and evaluation metrics are provided in Sections B.1, B.2, B.3 in S1 Text.

**Fig 3:**
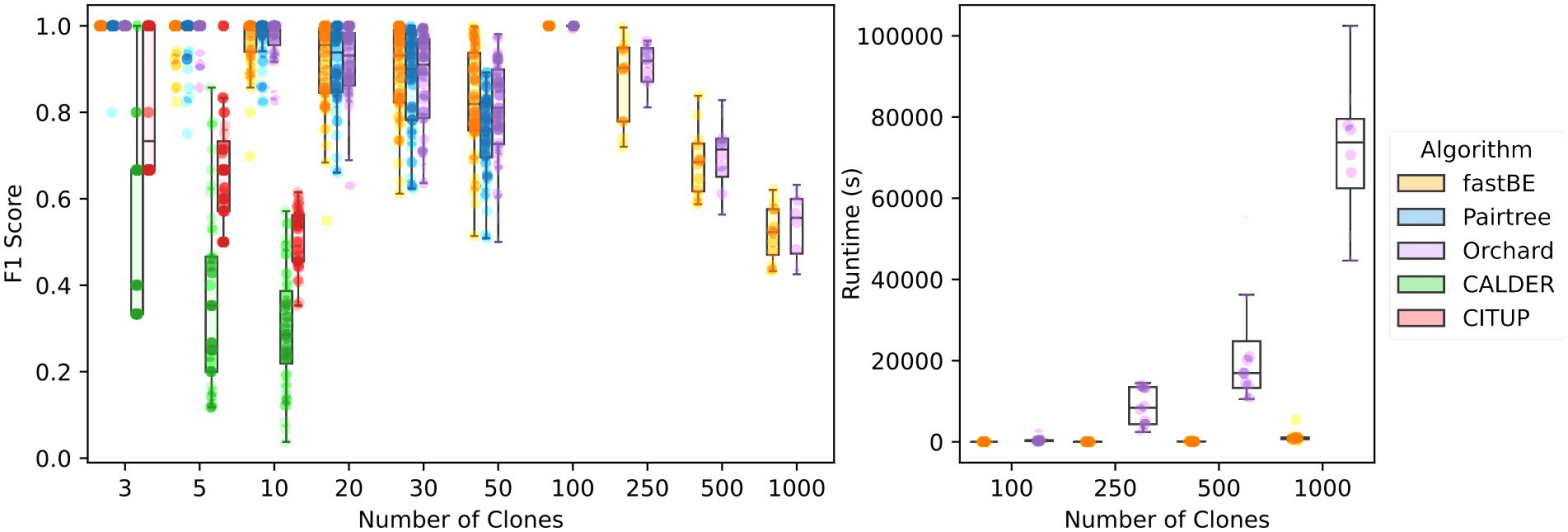
The accuracy of reconstructing pairwise relationships for the phylogenetic trees inferred by *fastBE*, Pairtree [11], Orchard [12], CALDER [10], and CITUP [7] on simulated data. **(Left)** The F1-score versus the number of clones. **(Right)** The wall-clock runtime on instances with ≥100 clones versus the number of samples. Methods that did not scale to instances with many clones are excluded from the plot.

On simulated instances with few clones (*n* = 3, 5, 10) and samples (*m* = 5, 10, 25), all algorithms terminated in under 24 hours on the majority of the 108 simulated instances (*fastBE*: 108/108, Pairtree: 108/108, Orchard: 108/108, CALDER: 106/108, CITUP: 107/108). *fastBE*, Pairtree, and Orchard accurately recovered pairwise relationships in this setting (Fig 3 and S5 Fig), with *fastBE*, Pairtree, and Orchard performing nearly identically for the *n* = 10 clone setting in terms of mean F1-score (*fastBE*: 0.965, Pairtree: 0.972, Orchard: 0.965).

In contrast, CITUP and CALDER struggled to accurately reconstruct pairwise relationships (Fig 3 and S5 Fig) when there were 5 or more clones. In terms of recovering the ground truth usage matrix *U* and frequency matrix *F*, *fastBE* significantly outperformed CITUP and CALDER, while performing similarly to Pairtree and Orchard (S6 Fig).

On simulated instances with a modest number of clones (*n* = 20, 30, 50) and samples (*m* = 25, 50), CITUP and CALDER were unable to terminate on the majority of the simulated instances within 24 hours—consistent with the findings of [11, 12]—and were excluded from our evaluation. On instances with *n* = 20, 30 clones, *fastBE*, Pairtree, and Orchard performed similarly in terms of reconstructing pairwise relationships (Fig 3 and S7 Fig). However, on instances with *n* = 50 clones, *fastBE* and Orchard outperformed Pairtree (mean F1 *fastBE*: 0.825, Pairtree: 0.749, Orchard: 0.805) in reconstructing pairwise relationships (S13 Fig). In terms of recovering the ground truth usage matrix *U* and frequency matrix *F*, all methods performed quite well in recovering *F*, but Pairtree was less accurate in recovering *U* and *F* when the number of clones was large (S8 Fig). Finally, *fastBE* was an order of magnitude faster than Pairtree and Orchard, running for an average of 1.21 seconds on instances with *n* = 50 clones (S13 Fig).

In the regime with a large number of clones (*n* = 100, 250, 500, 1000) and samples (*m* = 50, 100), only *fastBE* and Orchard were able to terminate within a 24 hour time limit. On these instances, *fastBE* and Orchard perform nearly identically in terms of recovering ground truth pairwise relationships (mean F1 *fastBE*: 0.773, Orchard: 0.783). The methods also had similar performance in recovering the ground truth usage matrix *U* and frequency matrix *F* (S8 Fig). In terms of runtime, however, *fastBE* was several orders of magnitude faster than Orchard (Fig 3 and S13 Fig). For example, *fastBE* took a mean of 1229.8 seconds to run on instances with *n* = 1000 clones and terminated on all such instances, whereas Orchard took a mean of 71749.1 seconds and terminated on 19/24 such instances when allotted 48 hours and a dedicated 32-core processor.

We also found that *fastBE* was more *sample efficient* than other methods, requiring fewer samples to recover the ground truth clonal relationships. In particular, the pairwise reconstruction accuracy strictly improved for *fastBE*, Pairtree, and Orchard as the number of samples increased (Fig 3 and S12 Fig), while this was not necessarily the case for CALDER and CITUP (S19 and S10 Figs). However, the reconstruction accuracy improved for *fastBE* more quickly than Pairtree and Orchard (Fig 3 and S12 Fig) as the number of samples increased, and *fastBE* obtained near perfect recovery (median F1: 0.987) with the number of samples *m* ≥ 50 and the number of clones *n* < 100. This observation led us to investigate the reconstruction accuracy as the ratio of samples to clones increased. Interestingly, we observed a sharp transition in pairwise reconstruction accuracy for *fastBE* as the ratio of samples to clones approached one (S11 Fig).

### 3.3 Evaluation of phylogeny constrained clustering with *fastBE* on simulated data

We compared our phylogeny constrained mutation clustering algorithm (Section 2.4) to both the mutation clustering algorithm in Orchard [12] and the method PyClone-VI [29] on a low-coverage simulated dataset. To perform our evaluation, we created ground truth mutation clusters by simulating a clone tree T and a usage matrix *U*, assigning each mutation to one of the clones in T. To construct each simulated instance, we generated a clone tree T and usage matrix *U*, computed the frequency matrix *F* = *UB*, and sampled both variant and non-variant reads for each mutation from *F* at 20× coverage. By construction, the variant read sampling frequency was identical for mutations belonging to the same clone and sample. Complete details describing the simulations are provided in Sections B.1 in S1 Text.

To infer mutation clusters, we first built phylogenetic trees from the mutation-level variant and total read count matrices for *fastBE* and Orchard. Using these inferred phylogenies, we then applied our mutation clustering algorithm (Section 2.4) and Orchard’s mutation clustering algorithm, respectively, passing in the ground truth number of clusters. For PyClone-VI, we simply passed in the mutation-level variant and total read count matrices to obtain mutation clusters. To evaluate the quality of the inferred clusterings, we applied two metrics, the *adjusted rand index* (ARI) and the *normalized mutual information score* (NMI), to the ground truth and inferred mutation clusters. Across all simulated settings, *fastBE* had both the highest mean ARI and NMI, with Orchard falling in second place (S14 Fig). Interestingly, on several instances Orchard exactly inferred the true mutation clusters, however, Orchard’s performance was quite variable, sometimes attaining an ARI of approximately 0. PyClone-VI, though state-of-the-art for mutation clustering, performed worst on all simulated settings, illustrating the utility of using a phylogeny-aware approach.

### 3.4 Analysis of B progenitor acute lymphoblastic leukemia patient samples

We applied *fastBE* to infer phylogenetic trees from multi-sample bulk DNA sequencing data of fourteen patients with B progenitor acute lymphoblastic leukemia (B-ALL) [16]. This dataset was generated by whole exome sequencing (≈ 200× coverage) of tissue samples from fourteen B-ALL patients at both diagnosis and relapse time points. Both diagnosis and relapse samples were subsequently grafted onto immunodeficient mice, generating additional patient-derived xenografts further sequenced using targeted sequencing. Using orthogonal copy number information, mutations were excluded from downstream analysis if they did not lie in copy number neutral regions [16]. Otherwise, all mutations were included for downstream analysis, regardless of their driver or passenger status. Taking both patient and derived xenograft samples together, this process resulted in fourteen patient samples containing a median of 42 (min: 13, max 90) samples and a median of 42 mutations (min: 17, max 293) per patient.

We inferred phylogenies using *fastBE*, Pairtree, and Orchard directly from the mutation-level variant and total read counts provided by Pairtree [11]. While *fastBE* and Orchard were successfully able to infer phylogenies on all fourteen patient samples, Pairtree failed to terminate on the two largest samples, SJETV010 and SJBALL022610, which contained 130 and 293 mutations respectively. On the remaining samples, *fastBE* was substantially faster than both Orchard and Pairtree (S15 Fig), terminating in less than 80 seconds on all samples. On all 14 of the patient samples, each of *fastBE*, Pairtree, and Orchard inferred distinct trees. However, directly quantifying the similarity of the inferred trees was challenging due to long chains of mutations, many of which can be arbitrarily reordered without affecting the reconstruction accuracy.

We quantified the differences between the phylogenetic trees inferred by *fastBE*, Pairtree, and Orchard by examining two metrics of concordance with the observed data: the *frequency matrix estimation error* and violations of the *sum condition* (Eq (11)) [5–7, 24]. For all methods, the normalized frequency matrix estimation error $\frac{1}{mn}{\left\|F-\hat{F}\right\|}_{1}$ (B.3 in Supplementary Text A) was less than 0.1 in all cases (S16 Fig). However, the trees inferred by *fastBE* had substantially lower frequency matrix estimation error (mean error *fastBE*: 1.2 × 10^−3^, Pairtree: 2.1 × 10^−3^, Orchard: 1.7 × 10^−3^), suggesting a better fit to the observed mutational frequencies. The *sum condition* requires that the frequency *F*_*i*,*j*_ of a mutation *j* gained at a clone in sample *i* is greater than or equal to the sum ∑_*k*∈*C*(*j*)_ *F*_*i*,*k*_ of the frequencies of the mutations gained at the clone’s children. The sum condition follows from the perfect phylogeny assumption, which states that each mutation is gained at most once and never lost. Consequently if a mutation is present in a clone, then the mutation is present in all the clone’s children. Importantly, all of the methods benchmarked [7, 10–12] make the perfect phylogeny assumption, and thus if the frequency matrix is correctly measured, the inferred trees should satisfy the sum condition. For a sample *i* and mutation *j*, we define the *violation* *V*_*i*,*j*_ = max{∑_*k*∈*C*(*j*)_ *F*_*i*,*k*_ − *F*_*ij*_, 0} of the sum condition. The *total violation* *V* of the sum condition is the sum *V* = ∑_*i*,*j*_ *V*_*i*,*j*_ of the violations over all samples and mutations. We found that the phylogenies inferred by *fastBE* had a lower total violation *V* (mean *V* of 23.9 over 14 patients) compared to both Pairtree (mean *V* of 30.1 over 12 patients) and Orchard (mean *V* of 29.4 across all 14 patients). The reduced violation of the sum condition demonstrated by *fastBE* also held across individual experiments and mutations (S17 and S18 Figs).

Next, we inferred mutation clusters on the *fastBE* and Orchard phylogenies using the phylogeny-aware clustering algorithms described in (Section 2.4) and [12] respectively. Since both of these methods require the number of clusters *k* as input to the method, we performed a manual *elbow* analysis to select the number of clusters (S19 Fig), providing the selected number of clusters *k* to both methods. Interestingly, both methods inferred relatively similar mutation clusters, obtaining a mean ARI of 0.53 (S20 Fig), though this varied between samples. For example, while for the SJBALL022612 patient sample, *fastBE* and Orchard inferred nearly identical mutation clusters, for SJETV010 the clusters inferred by both methods varied drastically. To evaluate the mutation clusters output by both methods, we computed the mutation cluster distortion $\sum_{k=1}\sum_{j\in C_{i}}{\left\|F_{:,j}-c_{j}\right\|}_{1}$ for the clusters output by each method. While quite similar overall, *fastBE* had a slightly lower distortion on average, with an average distortion 77.2 as opposed to 80.1 (S21 Fig).

**Fig 4:**
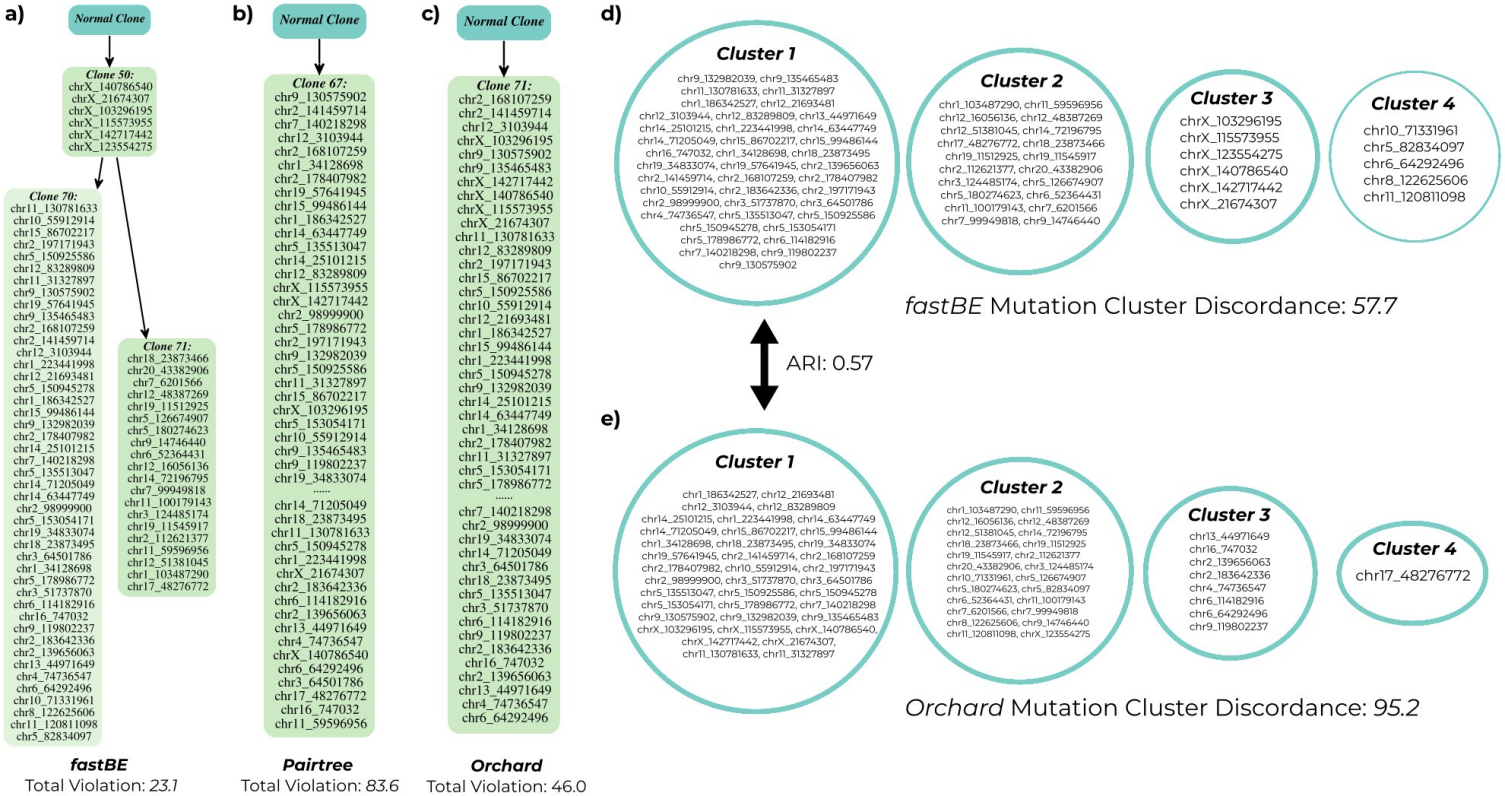
The mutation phylogenies inferred by **a)** *fastBE*, **b)** Pairtree [11], and **c)** Orchard [12] for the patient sample SJBALL022613 from multi-sample bulk DNA sequencing data of fourteen patients with B progenitor acute lmyphoblastic leukemia [16]. For ease of visualization, we collapsed vertices with out-degree 1 into a single node, preserving the order of mutations as they appeared in the original tree. The mutation clusters inferred using the phylogeny-aware clustering algorithms of *fastBE* **d)** and Orchard **e)** for the patient sample SJBALL022613.

Qualitatively, we observed differences between the trees inferred by *fastBE* as compared to those inferred by Pairtree and Orchard. For example, Pairtree and Orchard (median average depth Pairtree: 15.3, Orchard: 17.04) tended to infer deeper trees as compared to *fastBE* (median average depth: 10.2), and also tended to place mutations on long chains of degree two vertices. As a concrete example, we took a closer look at the phylogenetic trees inferred for patient sample SJBALL022613. For this patient sample, which contained 20 samples and 72 mutations, both the Orchard and Pairtree inferred a linear phylogeny, suggesting linear evolution [48]. In contrast, the *fastBE* phylogeny branched into two distinct lineages, suggesting a branched evolution (Fig 4A–4C). The phylogeny inferred by *fastBE* had lower total violation of the sum condition compared to the phylogenies inferred by Pairtree and Orchard, though Orchard had subsantially less total violation than Pairtree. The mutation clusters inferred by *fastBE* and Orchard were similar, with an ARI of 0.57, but contained notable differences (Fig 4D and 4E). For example, the mutation clusters inferred by *fastBE* were more uniform in size, and placed all mutations on the X-chromosome into a single mutation cluster off the root of the phylogeny. While Orchard also inferred that the X-chromosome mutations occurred early in the tumor’s evolution, it spread these mutations across multiple clusters. Finally, the clusters inferred by *fastBE* had lower mutation cluster distortion $\sum_{k=1}\sum_{j\in C_{i}}{\left\|F_{:,j}-c_{j}\right\|}_{1}$ than those inferred by Orchard (Fig 4D and 4E).

### 3.5 Analysis of patient-derived colorectal cancer models

We compared *fastBE* to Pairtree and Orchard on two patient-derived xenograft models of colorectal cancer, POP66 and CSC28 [49], from which multiple bulk samples underwent whole-exome sequencing. The POP66 model contained eight samples collected in the parent tumor (P0), first generation xenograft (G0), and regrowth xenografts, and 25 mutation clusters were inferred across these samples in [49]. The CSC28 model consisted of four samples collected in the first generation (G0) and regrowth xenografts, and 11 mutation clusters were inferred across these samples in [49]. Due to the high read depth of whole-exome sequencing (≈50× sequencing), we inferred phylogenies directly from the mutation-level variant and total read counts rather than the previously reported mutation clusters. Following the original publication [49], we excluded mutations that were contained in copy number aberrations in any of the samples, and used the copy number corrected mutation-level variant and total read counts provided by [49].

We found that the phylogenetic trees inferred by *fastBE* were quite different from those inferred by Pairtree and Orchard in terms of both their implied pairwise relationships and overall structure (S22 and S23 Figs). For example, while both the *fastBE* and Orchard CSC28 phylogenies had the mutation ORG13G occurring off the root, only the *fastBE* CSC28 phylogeny implied a polyclonal tumor origin. Further, the CSC28 phylogeny inferred by *fastBE* was substantially less deep than those inferred by Pairtree and Orchard. A similar story appears for the POP66 phylogenies, where the differences are even further exaggerated due to the large number of mutations.

Finally, we quantified the frequency matrix estimation error and total violation of the sum condition in the phylogenetic trees inferred by *fastBE*, Pairtree, and Orchard. We found that *fastBE* had both the lowest frequency matrix estimation error and total violation of the sum condition, though Orchard outperformed Pairtree by a large margin (S24 Fig).

## 4 Discussion

We defined a linear optimization problem, the *ℓ*_1_-VAFRP, a subproblem of the NP-complete *ℓ*_1_-VAFFP. By exploiting the special structure of the matrices which appear in this regression problem, we derived an algorithm which runs in O(mnd) time where *m* is the number of samples, *n* is the number of clones, and *d* is the depth of the input tree T, obtaining asymptotic and empirical speedups over state-of-the-art linear programming solvers. Using our regression algorithm, we developed a method *fastBE* for the *ℓ*_1_-VAFFP which scales to large, multi-sample bulk DNA sequencing datasets. While *fastBE* serves as a practically useful tool for phylogenetic inference, we also believe our *ℓ*_1_-regression algorithm and structured regression model is of independent interest, and will serve as a useful tool for the development of other algorithms for phylogenetic inference from multi-sample bulk DNA sequencing data.

There are several limitations of the present approach, which are directions for future work. On the theoretical side, it is an open question whether the time complexity of our regression algorithm can be improved from the current O(mnd) time to the optimal O(mn) time, which is the size of the input. On the practical side, extending our model and regression algorithm to additional classes of evolutionary models is desirable. Here, we analyzed the simplest case of single nucleotide variants in copy neutral regions. Accounting for copy number heterogeneity by replacing the VAF with either the cancer cell fraction [50–52] or the descendant cell fraction [53], could improve the performance of our method on real datasets. Furthermore, using evolutionary models that allow for mutation loss—e.g., the Dollo model [5, 54], or generalizations [55, 56]—is a challenging future direction. Finally, extending or applying *fastBE* to infer repeated evolutionary trajectories [57–60] across patients may extend the utility of *fastBE* beyond the single-patient setting.

## Supporting information

S1 Text Supplementary text file (PDF) containing supplementary methods, results, and proofs. (PDF)

S1 Fig Relative runtime analysis of our *ℓ*_1_ regression algorithm and LP solvers. (TIFF)

S2 Fig Absolute runtime analysis of our *ℓ*_1_ regression algorithm and LP solvers. (TIFF)

S3 Fig Relative runtime analysis of warm versus cold starting our *ℓ*_1_ regression algorithm. (TIFF)

S4 Fig Absolute runtime analysis of warm versus cold starting our *ℓ*_1_ regression algorithm. (TIFF)

S5 Fig FPR and FNR of inferring pairwise relations for ≤ 10 clone and ≤ 25 sample simulated instances. (TIFF)

S6 Fig Inferred matrix error for ≤ 10 clone and ≤ 25 sample simulated instances. (TIFF)

S7 Fig FPR and FNR of inferring pairwise relations for ≥ 20 clone simulated instances. (TIFF)

S8 Fig Inferred matrix error for ≥ 20 clone simulated instances. (TIFF)

S9 Fig FPR and FNR of inferring pairwise relations versus ratio of samples to clones with ≤ 10 clones and ≤ 25 samples. (TIFF)

S10 Fig FPR and FNR of inferring pairwise relations versus number of samples with ≤ 10 clones. (TIFF)

S11 Fig FPR and FNR of inferring pairwise relations versus ratio of samples to clones with between 20 and 100 clones. (TIFF)

S12 Fig FPR and FNR of inferring pairwise relations versus the number of samples with between 20 and 100 clones. (TIFF)

S13 Fig Runtime analysis of *fastBE*, Pairtree, and Orchard. (TIFF)

S14 Fig ARI and NMI of inferring mutation clusters. (TIFF)

S15 Fig Runtime analysis for B-ALL patient phylogenies. (TIFF)

S16 Fig *ℓ*_1_ matrix error for B-ALL patient phylogenies. (TIFF)

S17 Fig Total violation of the sum condition for B-ALL patient phylogenies. (TIFF)

S18 Fig Per mutation total violation of the sum condition for B-ALL patient phylogenies. (TIFF)

S19 Fig B-ALL mutation clustering distortion versus the number of clusters. (TIFF)

S20 Fig B-ALL mutation clustering ARI between *fastBE* and Orchard clusterings. (TIFF)

S21 Fig B-ALL mutation clustering distortion difference for *fastBE* and Orchard clusterings. (TIFF)

S22 Fig Inferred phylogenies for CSC28. (TIFF)

S23 Fig Inferred phylogenies for POP66. (TIFF)

S24 Fig Sum condition violation and frequency matrix estimation error on POP66 and CSC28. (TIFF)

S25 Fig F1 score and runtime analysis of *fastBE* on imperfect phylogenies. (TIFF)
