# Supplementary material for: A regression based approach to phylogenetic reconstruction from multi-sample bulk DNA sequencing of tumors
Source: PLoS Comput Biol. 2024 Dec 4;20(12):e1012631. doi: 10.1371/journal.pcbi.1012631 (PMC11661639; doi:10.1371/journal.pcbi.1012631)
Supplement: S1 Text — (PDF) [file pcbi.1012631.s001.pdf]

S1 Text: A regression based approach to phylogenetic reconstruction from multi-sample bulk DNA sequencing of tumors

Henri Schmidt[1] and Benjamin J. Raphael[1,†]

[1]Department of Computer Science, Princeton University, NJ, USA
[†]Correspondence: braphael@princeton.edu

A Supplementary Results

Proof of Proposition 1. Suppose the theorem holds for all $k < n$. Clearly, the theorem holds for the base case when $n = 1$.

$(i) \Rightarrow (ii)$: Let $\mathcal{T}$ be the n-clonal tree associated with B. Consider a relabeling of the clones of $\mathcal{T}$ by their preorder traversal index during a depth first search of $\mathcal{T}$ starting at the root. Then, $r(\mathcal{T})$ is assigned index 1 and the children of $r(\mathcal{T})$ are assigned indices $i_1 < \ldots < i_k$ where k is the number of children of $r(\mathcal{T})$. Let B' be the clonal matrix associated with $\mathcal{T}$ under this relabeling. Then, $B'_{i,1} = 1$ and $B'_{1,j} = 0$ for all $j \in \{2, \ldots, n\}$ since all clones contains the mutation 1 and the root only contains the mutation 1. Let $B_1, \ldots, B_k$ be defined recursively as the clonally canonical matrices associated with the subtrees rooted at the children $i_1, \ldots, i_k$ of $r(\mathcal{T})$, which exist by the inductive hypothesis. Then, B' is clonally canonical with blocks $B_1, \ldots, B_k$.

To see this, observe that all vertices $l \in V(\mathcal{T}_{i_j})$ satisfy $i_{j-1} < l < i_{j+1}$ as we performed a depth first traversal to relabel $\mathcal{T}$. This implies that the sub-matrix $B'[l_i : l_{i+1} - 1, l_i : l_{i+1} - 1]$ is equal to B_i, where we set $l_{k+1} = n + 1$. Since B_i are clonally canonical, this completes this direction of the proof.

The equivalence of (i) and (iii) follows from the proof of Lemma 1 in [1]. It follows that (ii) implies (i) as clonally canonical matrices trivially satisfy (iii). ∎

Proof of Proposition 2. By Lemma 1, the clonal matrix inverse $B^{-1} = (I - A)$ and has exactly $2n$ non-zero entries. As such, we can compute the products $B^{-1}v$ and $v^T B^{-1}$ in $O(n)$ time as the tree $\mathcal{T}$ defines the indices of non-zero entries.

Next, consider a clonally canonical matrix B with clonally canonical blocks $B_1, \ldots, B_k$ with dimensions $n_1, \ldots, n_k$ such that $\sum_{i=1}^{k} n_i = n - 1$. Define the coordinates $l_i = 2 + \sum_{j=1}^{i} n_j$ and *row vectors* $v^{(i)} = (v_{l_{i-1}}, \ldots, v_{l_i} - 1)$, such that $v^T = (v_1, v^{(1)}, \ldots, v^{(k)})$. Then, by the properties of block matrix multiplication,

$$v^T B = (\mathbb{1}^T v, v^{(1)} B_1, \ldots, v^{(k)} B_k).$$

However, since $B_1, \ldots, B_k$ are also clonally canonical, we have that the first entry of $v^{(i)} B_i$ is the sum of the entries of $v^{(i)}$, implying that

$$\mathbb{1}^T v = v_1 + [v^{(1)} B_1]_1 + \ldots + [v^{(k)} B_k]_1.$$

Therefore, after computing the k products $v^{(i)} B_i$, we can compute the $v^T B$ with only $k + 1$ additional operations.

Assuming inductively that the number of operations to compute the product $u^T B'$ for any clonally canonical matrix B' of size less than $n' < n$ is bounded by $2n' - 1$, the total number of operations to compute $v^T B$ is at most

$$(k + 1) + (2n_1 - 1) + \ldots + (2n_k - 1) = k + 1 + 2(n - 1) - k = 2n - 1,$$

proving that $v^T B$ is computable in $O(n)$ time.

To see that the product Bv can also be efficiently computed, treat $v^{(i)}$ as a *column* vector. Then, by the properties of block matrix multiplication,

$$Bv = (v_1, v_1 \mathbb{1} + B_1 v^{(1)}, \ldots, v_1 \mathbb{1} + B_k v^{(k)}).$$

Therefore, after computing the k products $B_i v^{(i)}$ with an offset term v_1, we obtain Bv with only a single operation, proving that Bv is computable in $O(n)$ time by a similar argument as above. ■

Proof of Lemma 1. Observe that $A^k_{i,j} = 1$ if and only if j is an ancestor of i in $\mathcal{T}$ at tree distance k from i. Then,

$$B = A^0 + A^1 + A^2 + \cdots + A^n = I + A + A^2 + \cdots = (I - A)^{-1}$$

where the first equality follows from the observation above. The second equality from the observation that A^k is the zero matrix when $k > n$. ■

Proof of Corollary 2. The first part of the corollary follows Lemma 4 of El-Kebir et al. [1]. The second part of the corollary follows from observing that

$$x_i = \begin{cases} 1 & \text{if } \sum_{j \in C(i)} f_j - f_i > 0, \\ 0 & \text{otherwise.} \end{cases}$$

is a feasible solution to the DPTLP in Theorem 2. ■

B Supplementary Methods

B.1 Simulation details

Our simulation procedure loosely follows the steps outlined in AncesTree [1]. Briefly, we simulate an n-clonal tree $\mathcal{T}$, a m-by-n usage matrix U, and assign each of the k mutations to one of the n clones. Then, for each sample-mutation pair, we sample total and variant read counts.

In more detail, we constructed two sets of simulated instances, referred to as the *small* and *large* simulated instances respectively. The small simulated instance is defined by the following set of five parameters:

- $n \in \{3, 5, 10, 20, 30, 50\}$: the number of clones,
- $m \in \{5, 10, 25, 50\}$: the number of samples,
- $k \in \{500\}$: the number of mutations,
- $d \in \{40\}$: the read depth,
- $s \in \{1, \ldots, 11, 12\}$: the random seed,

yielding a total of 288 small simulated instances. The large simulated instance is defined by the following set of parameters:

- $n \in \{100, 250, 500, 1000\}$: the number of clones,
- $m \in \{50, 100\}$: the number of samples,
- $k \in \{2000\}$: the number of mutations,
- $d \in \{40\}$: the read depth,
- $s \in \{0, 1, 2, 3, 4, 5\}$: the random seed,

yielding a total of 48 large simulated instances.

For a fixed set of parameters (n, m, k, d, s), we perform the following sequence of steps to create our simulation:

1. Randomly sample an n-clonal tree $\mathcal{T}$ with root vertex 0 from the set of all n-clonal trees using Wilson's algorithm.
2. Randomly assign each of the k mutations to one of the n clones to obtain a (surjective) map from mutations to clones $\psi : [k] \to [n]$.
3. Randomly sample a usage matrix U row-by-row:
   (a) For each row $i \in [m]$, sample the number of non-zero entries $l_i \sim \text{Uniform}(1, \ldots, n)$.
   (b) For row $i \in [m]$, select the indices of the l_i non-zero usage proportions uniformly at random. Then, select the value of the l_i non-zero proportions from a Dirichlet distribution.
4. Set the ground-truth frequency matrix $F = UB_{\mathcal{T}}$.
5. Sample the total read counts $T_{i,j} \sim \text{Poisson}(d)$ for all samples $i \in [m]$ and mutations $j \in [k]$.
6. Sample the variant read counts $V_{i,j} \sim \text{Binomial}(T_{i,j}, F_{i,\psi(j)})$ for all samples $i \in [m]$ and mutations $j \in [k]$.

B.2 Running phylogenetic methods

To run *fastBE*, CITUP [2], CALDER [3], Pairtree [4], and Orchard [5] on the simulations, we passed in the variant read counts V, the total read counts T, and the mutation to clone mapping $\psi : [k] \to [n]$. For *fastBE* and CALDER [3], we first estimated the frequency matrix $\hat{F}$ as

$$\hat{F}_{ij} = \frac{\sum_{k \in \psi^{-1}(j)} V_{ik}}{\sum_{k \in \psi^{-1}(j)} T_{ik}}.$$

When running CITUP [2], we used the quadratic integer programming version of the method and set the max jobs equal to the number of compute cores. For CALDER, we used the ℓ_1 objective and default parameters. For Pairtree [4], we used the default parameters and explicitly set the number of threads equal to the number of available compute cores. Since Pairtree outputs a posterior distribution over trees rather than a single tree, we select the most likely tree output by their tool. For Orchard [5], we used the recommended settings outlined in their Github repository. Similarily, we select the most likely tree output by their tool.

To benchmark the regression algorithm described in (Section 2.2.3) against the linear programming based approaches implemented by the solvers Gurobi [6] and CPLEX [7], we constructed larger simulated instances with $n \in \{250, 500, 750, 1000\}$ clones, $m \in \{50, 100, 200, 500\}$ samples, and $k \in \{500, 1000, 2000, 4000\}$ mutations at a read depth of $d = 50$. For each of the three regression algorithms, we passed in the ground truth frequency matrix F and clonal matrix $B_{\mathcal{T}}$, and then measured both the wall-clock and (internal) solver time to infer the usage matrix U which minimizes the ℓ_1 error $\|F - UB_{\mathcal{T}}\|_1$. All simulations and method evaluations were performed on the computing cluster provided by the Princeton University Department of Computer Science. Each simulated instance and method evaluation was performed independently on cluster nodes with 8 GB of memory and 16 compute cores.

B.3 Evaluation metrics

Our evaluation procedure is philisophically similar to [1–3, 8, 9] in that we believe there is a single ground truth clone tree and usage matrix, and that the goal of factorization is to recover this ground truth. In contrast, the evaluation philosophy of Pairtree [4] believes that the inherent ambiguity leads to many, equally plausible clone trees and usage matrices, and that the goal of factorization is to learn this set (or distribution) of clone trees and usage matrices. Therefore, we evaluate methods with respect to their deviation from the ground truth clonal tree $\mathcal{T}$, usage matrix U, and frequency matrix F.

We first measure the deviation between the inferred clonal tree $\hat{\mathcal{T}}$ and the ground truth clonal tree $\mathcal{T}$. To perform this measurement, we compute the set of ancestral relationships for each tree, and measure how similar these sets are to each other. Formally, we define the set of ancestral relationships as follows,

$$\text{rel}(\mathcal{T}) = \{(i, j) : i \text{ ancestor of } j \text{ in } \mathcal{T}\},$$

where we say (i, j) is a *positive* if $(i, j) \in \text{rel}(\mathcal{T})$ and a *negative* otherwise. Then, the total number of positive and negatives is,

$$\text{pos}(\mathcal{T}) = |\text{rel}(\mathcal{T})| \quad \text{and} \quad \text{neg}(\mathcal{T}) = n(n-1) - \text{pos}(\mathcal{T}),$$

and the false positive and false negative rate of $\hat{\mathcal{T}}$ predicting positive and negatives ancestral relationships is,

$$\text{fpr}(\mathcal{T}, \hat{\mathcal{T}}) = \frac{|\text{rel}(\hat{\mathcal{T}}) - \text{rel}(\mathcal{T})|}{\text{neg}(\mathcal{T})} \quad \text{and} \quad \text{fnr}(\mathcal{T}, \hat{\mathcal{T}}) = \frac{|\text{rel}(\mathcal{T}) - \text{rel}(\hat{\mathcal{T}})|.}{\text{pos}(\mathcal{T})}.$$

Similarly, we define precision, recall, and the F1-score, which is the harmonic mean of precision and recall.

We next measure the deviation between the inferred and ground truth usage and frequency matrices. In particular, given an estimated usage matrix $\hat{U}$ and frequency matrix $\hat{F}$, the matrix error is the normalized ℓ_1 error:

$$\frac{1}{mn}\|U - \hat{U}\|_1 = \frac{1}{mn}\sum_{i,j}|U_{ij} - \hat{U}_{ij}| \quad \text{and} \quad \frac{1}{mn}\|F - \hat{F}\|_1 = \frac{1}{mn}\sum_{i,j}|F_{ij} - \hat{F}_{ij}|.$$

When methods do not explicitly report $\hat{U}$ and $\hat{F}$, and instead only output $\hat{\mathcal{T}}$, to compute the matrix error we first need to estimate $\hat{U}$ and $\hat{F}$. To perform this estimation, we conservatively set $\hat{U}$ to be the minimizer of $\|F - \hat{U}B_{\hat{\mathcal{T}}}\|_1$ over all usage matrices $\hat{U}$ and then set $\hat{F} = \hat{U}B_{\hat{\mathcal{T}}}$. This estimation procedure ensures the matrix error $\|F - \hat{F}\|_1$ is minimized for the reported clonal tree.

B.4 *fastBE* details

While we outlined our greedy search algorithm *fastBE* in Section 2.3, we omitted a formal description of our procedure for choosing an order to add mutations and the efficient recomputation approach. First, to choose an order in which to add mutations, we use the *F-sum trick* introduced in Orchard [5]. In particular, we use the order $O = \{o_1, \ldots, o_n\}$ obtained by sorting the column sums of F in descending order. That is, the F-sum trick orders the mutations O such that,

$$\sum_{i=1}^{m} F_{i,o_1} \geq \sum_{i=1}^{m} F_{i,o_2} \geq \ldots \sum_{i=1}^{m} F_{i,o_n},$$

the intuition being that mutations with higher frequency should occur earlier in the evolutionary process. Second, by the latter part of Theorem 1, after the addition of a mutation to $\mathcal{T}$, it takes $O(mnd)$ time to recompute the score $L^*(F, B_{\mathcal{T}})$. Further, the adoption of a child of the parent can be implemented as an SPR move, also taking only $O(mnd)$

time to recompute the score $L^*(F, B_{\mathcal{T}})$ upon. Performing adoptions in the correct order then leads to the total time complexity of $O(mn^2 2^{\Delta^*} \tilde{d}^2)$ over all possible placements.

Rather than building and maintaining a single tree throughout the course of our search procedure, we maintain a pre-specified k partial trees, extending our greedy search to a *beam search*. In particular, at each iteration we perform the attachment procedure over all k trees, selecting the top k trees over all placements across all trees for the next iteration. The time complexity of the algorithm is only linear in k, and allows for *fastBE* to output multiple nearly optimal trees. On all simulated data analyzed in our manuscript, however, we set the *beam width*, $k = 1$, regressing to the greedy algorithm described in the main text.

When applying *fastBE* to clusters of mutations, as specified by a map $\psi : [k] \to [n]$ from mutation to cluster identity, we define the input frequency matrix $\hat{F}$ as

$$\hat{F}_{ij} = \frac{\sum_{k \in \psi^{-1}(j)} V_{i,k}}{\sum_{k \in \psi^{-1}(j)} T_{i,k}},$$

where $V_{i,j}$ and $T_{i,j}$ is the variant and total read count of mutation j in sample i. That is, the columns of $\hat{F}$ correspond to the average frequency of the mutations in each cluster.

B.5 Handling violations of the copy number neutral and infinite sites assumptions

When measured single-nucleotide mutations do not conform to either the copy number neutral or infinite sites assumptions, the matrix factorization model provided in Section 2.1, and consequently *fastBE*, is not immediately applicable. However, two common workarounds enable the correct application of the matrix factorization model and *fastBE*.

When a mutation is gained exactly once but is lost or impacted by copy number aberrations, twice the variant allele frequency (VAF) no longer correctly estimates the fraction of cells in the sample containing that mutation, also known as the *cancer cell fraction* (CCF). However, for the purpose of phylogenetic reconstruction, the CCF, and consequently the VAF, is not quite the correct quantity. Instead, one wants a phylogenetically aware quantity, the *descendent cell fraction* (DCF) [9] or *PhyloCCF* [10], which is defined as the fraction of cells descendant from the cell gaining a particular mutation. Estimating the frequency matrix F by replacing the VAF with the DCF, the matrix factorization model described in Section 2.1 then remains valid, and *fastBE* is readily applicable to this corrected frequency matrix. Estimation of the DCF is provided by DeCiFer [9].

Unfortunately, if a mutation is gained more than once, the DCF correction is no longer well-defined. To handle this more general setting, as well as arbitrary violations of the infinite sites and copy number neutral assumptions, we recommend removing mutations which violate the model assumptions. This conservative approach is taken in several works [11–13] applying the matrix factorization model to bulk DNA sequencing data. Importantly, the matrix factorization model then remains valid on the remaining mutations, allowing application of *fastBE*. If desired, after inferring a phylogeny on the subset of mutations satisfying the model assumptions, one can attach mutations back onto the inferred phylogeny using a tool such as DETOPT [13].

Of course, determining which mutations violate the model assumptions remains challenging. A conservative approach is to throw out mutations which provide a poor fit to the model. For example, one could first infer a phylogeny using *fastBE* with all the measured mutations. If all mutations fit the model assumptions, we would expect little or no error in the estimated frequency matrix. However, for mutations violating the model assumptions, we would expect to find some amount of error, allowing us to detect mutations which do not fit the model assumptions. Alternatively, one could remove all mutations which appear in regions which are not copy number neutral, as done in [12].

B.6 Robustness of *fastBE* to violations of the infinite sites assumption

We evaluated the robustness of *fastBE* to violations of the infinite sites assumption using simulated, imperfect phylogenies. We simulated 150 tumor phylogenies with $n = 50$ mutation loci, $s = 10, 20, 30$ samples, and $v = 0, 3, 5, 10, 20$ violations of the infinite sites assumption across $r = 1, \ldots, 10$ random seeds. To construct each phylogeny, we first built a rooted tree $\mathcal{T}$ with $n + v$ edges using the methodology described in Section B.1 of S1 Text. Then, we uniformly at random sampled a permutation of the edges $e_1, \ldots, e_n, \ldots, e_{n+v}$ of $\mathcal{T}$. For each edge e_i with $1 \leq i \leq n$, we labeled the edge e_i with a $0 \to 1$ gain at mutation locus i. For each edge e_i with $n + 1 \leq i \leq n + v$, we first flipped a fair coin to obtain X_i. If X_i was heads, we randomly sampled a mutation locus j not appearing in the subtree rooted of e_i, and labeled e_i with a $0 \to 1$ mutation at locus j. If X_i was tails, we randomly sampled a mutation locus j appearing as an ancestor of e_i, and labeled e_i with a $1 \to 0$ mutation at locus j. Using this phylogeny $\mathcal{T}$, we constructed a binary

$(n + v)$-by-n matrix B which recorded the presence or absence each mutation across the $n + v$ clones in $\mathcal{T}$. Next we selected a s-by-$(n + v)$ usage matrix U uniformly at random, and computed the resulting s-by-n frequency matrix $F = UB$. Finally, we sampled both variant and non-variant reads for each mutation from F at 40× coverage.

We measured the ability of *fastBE* to reconstruct the tree $\mathcal{T}'$ obtained by contracting $\mathcal{T}$ to the perfect phylogeny edges $e_1, \ldots, e_n$ across the 150 simulated instances. Interestingly, in the presence of only a small number of violations ($v = 3, 5, 10$) of the infinite sites assumption, *fastBE* accurately recovered the contracted tree $\mathcal{T}'$ (S25 Fig). Further, the performance degraded gracefully with the number of violations, suggesting that for only a small number of violations of the infinite sites assumption, application of *fastBE* is suitable. However, when the number of violations measured 40% of the total mutations ($v = 20$), *fastBE* performed less well in reconstructing the contracted tree $\mathcal{T}'$, especially when only a small number of samples were present. In terms of runtime, *fastBE* was unaffected by the total number of violations (S25 Fig).

References

1. El-Kebir, M., Oesper, L., Acheson-Field, H. & Raphael, B. J. Reconstruction of clonal trees and tumor composition from multi-sample sequencing data. *Bioinformatics* **31,** i62–i70 (June 15, 2015).
2. Malikic, S., McPherson, A. W., Donmez, N. & Sahinalp, C. S. Clonality inference in multiple tumor samples using phylogeny. *Bioinformatics* **31,** 1349–1356 (May 1, 2015).
3. Myers, M. A., Satas, G. & Raphael, B. J. CALDER: Inferring Phylogenetic Trees from Longitudinal Tumor Samples. *Cell Systems* **8,** 514–522.e5 (June 26, 2019).
4. Wintersinger, J. A. *et al.* Reconstructing Complex Cancer Evolutionary Histories from Multiple Bulk DNA Samples Using Pairtree. *Blood Cancer Discovery* **3,** 208–219 (May 5, 2022).
5. Kulman, E., Kuang, R. & Morris, Q. Orchard: building large cancer phylogenies using stochastic combinatorial search. *arXiv preprint arXiv:2311.12917* (2023).
6. Gurobi Optimization, LLC. *Gurobi Optimizer Reference Manual* 2023.
7. International Business Machines Corporation, LLC. *IBM ILOG CPLEX Optimization Studio Reference Manual* 2022.
8. Popic, V. *et al.* Fast and scalable inference of multi-sample cancer lineages. *Genome Biology* **16,** 91 (May 6, 2015).
9. Satas, G. & Raphael, B. J. Tumor phylogeny inference using tree-constrained importance sampling. *Bioinformatics* **33,** i152–i160 (July 15, 2017).
10. Grigoriadis, K. *et al.* CONIPHER: a computational framework for scalable phylogenetic reconstruction with error correction. *Nature Protocols,* 1–25 (2023).
11. Dobson, S. M. *et al.* Relapse-Fated Latent Diagnosis Subclones in Acute B Lineage Leukemia Are Drug Tolerant and Possess Distinct Metabolic Programs. *Cancer Discovery* **10,** 568–587 (Apr. 1, 2020).
12. Rehman, S. K. *et al.* Colorectal cancer cells enter a diapause-like DTP state to survive chemotherapy. *Cell* **184,** 226–242 (2021).
13. Wu, C. H. *et al. Determining Optimal Placement of Copy Number Aberration Impacted Single Nucleotide Variants in a Tumor Progression History* in *International Conference on Research in Computational Molecular Biology* (2024), 438–443.
