# Supplementary figures and images for: A regression based approach to phylogenetic reconstruction from multi-sample bulk DNA sequencing of tumors
Source: PLoS Comput Biol. 2024 Dec 4;20(12):e1012631. doi: 10.1371/journal.pcbi.1012631 (PMC11661639; doi:10.1371/journal.pcbi.1012631)

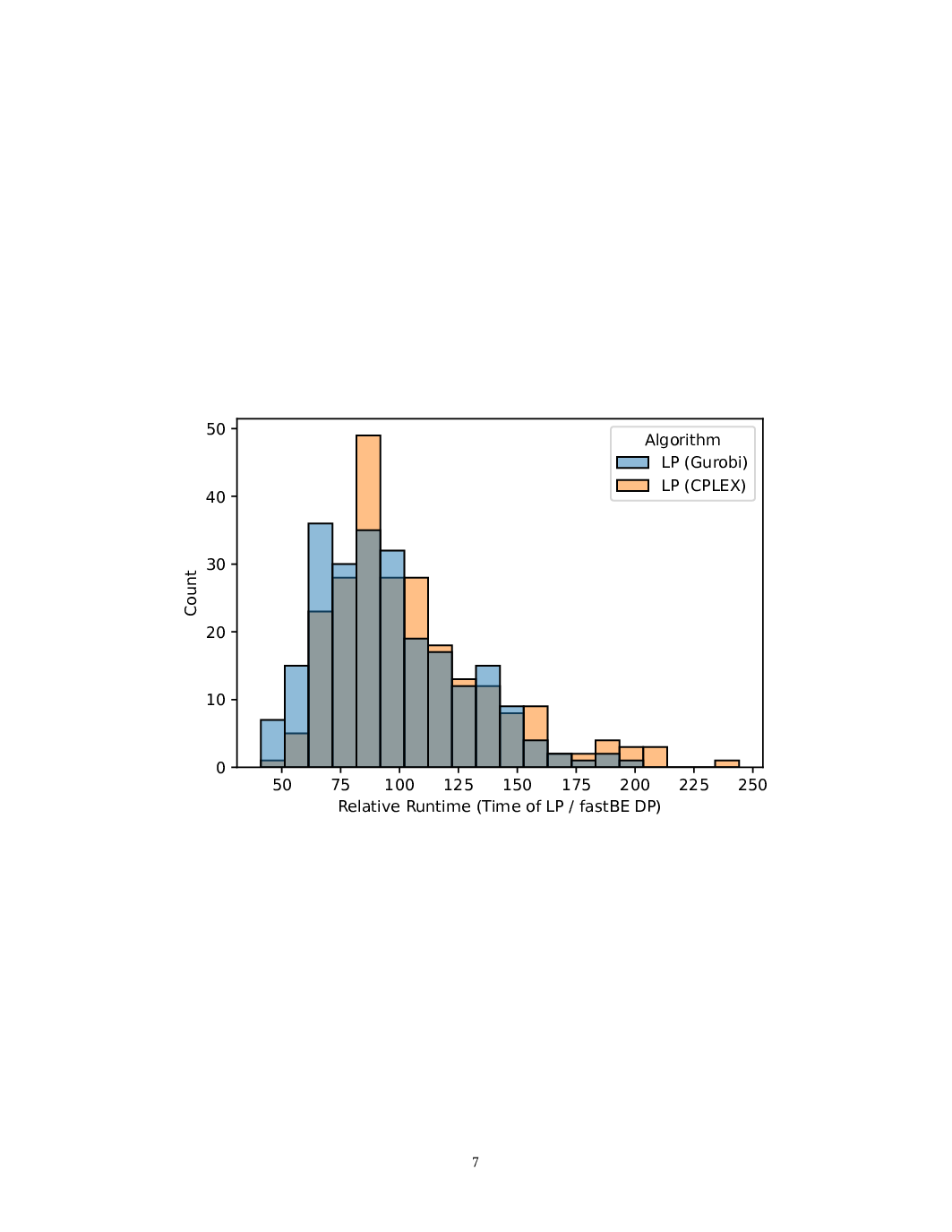

Supplement: S1 Fig — (TIFF) [file pcbi.1012631.s002.tiff]

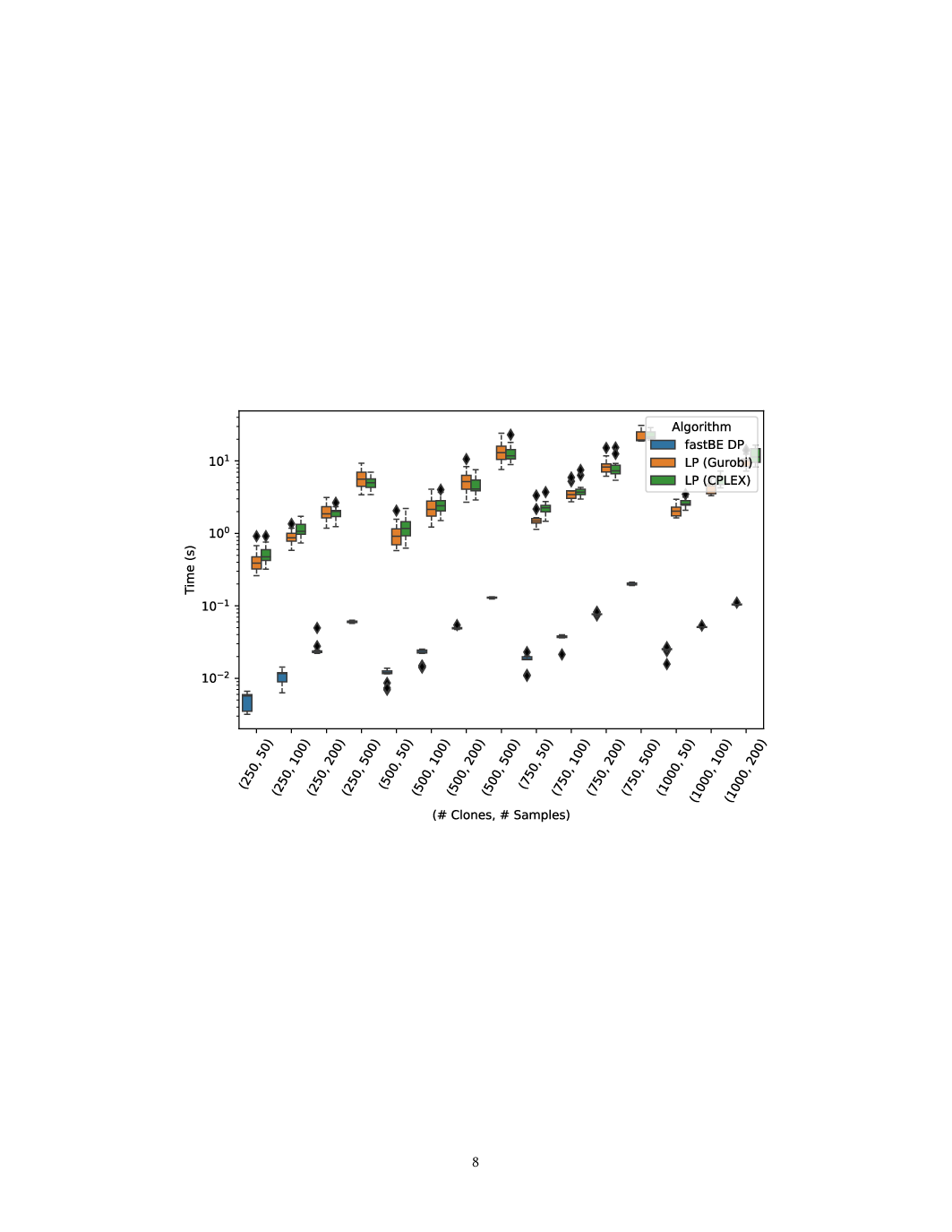

Supplement: S2 Fig — (TIFF) [file pcbi.1012631.s003.tiff]

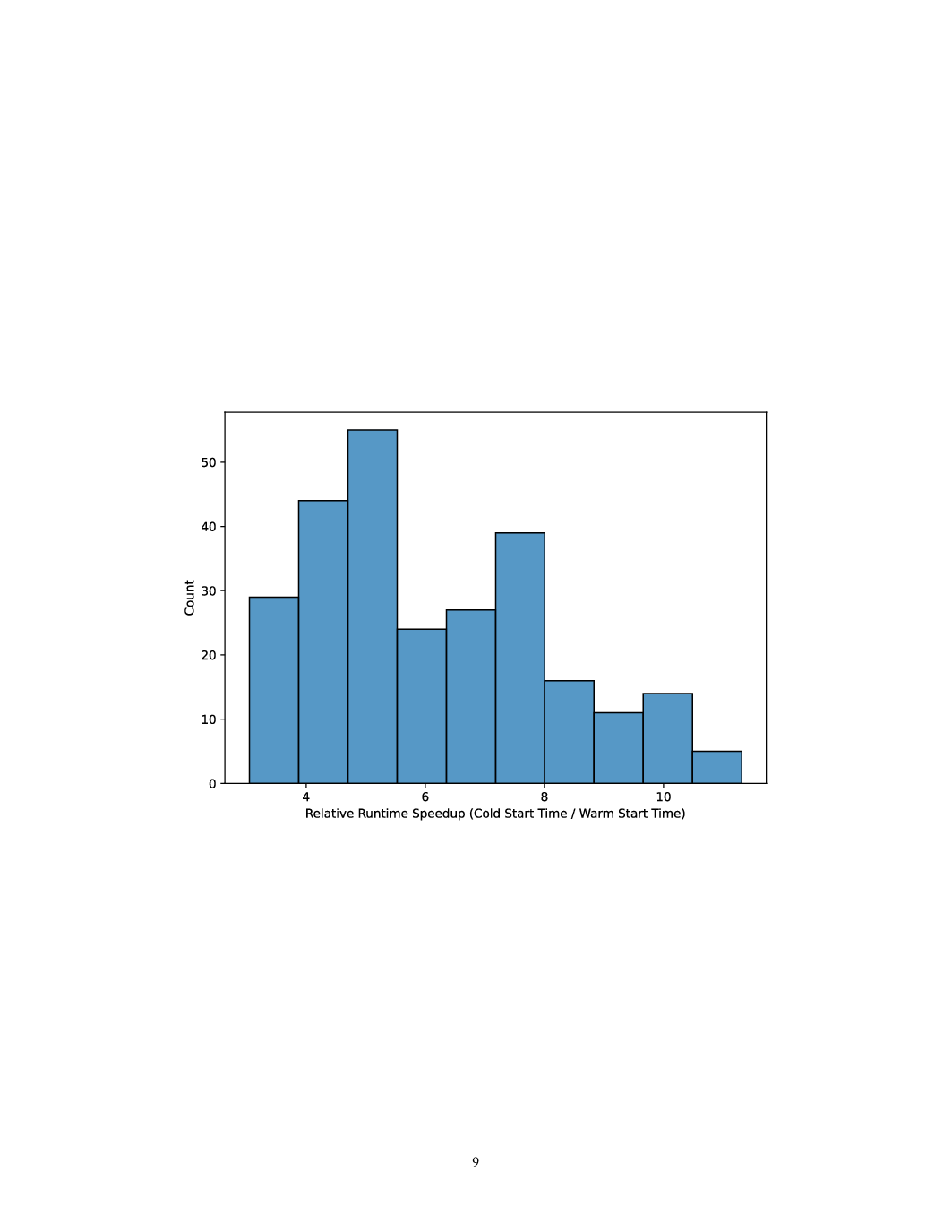

Supplement: S3 Fig — (TIFF) [file pcbi.1012631.s004.tiff]

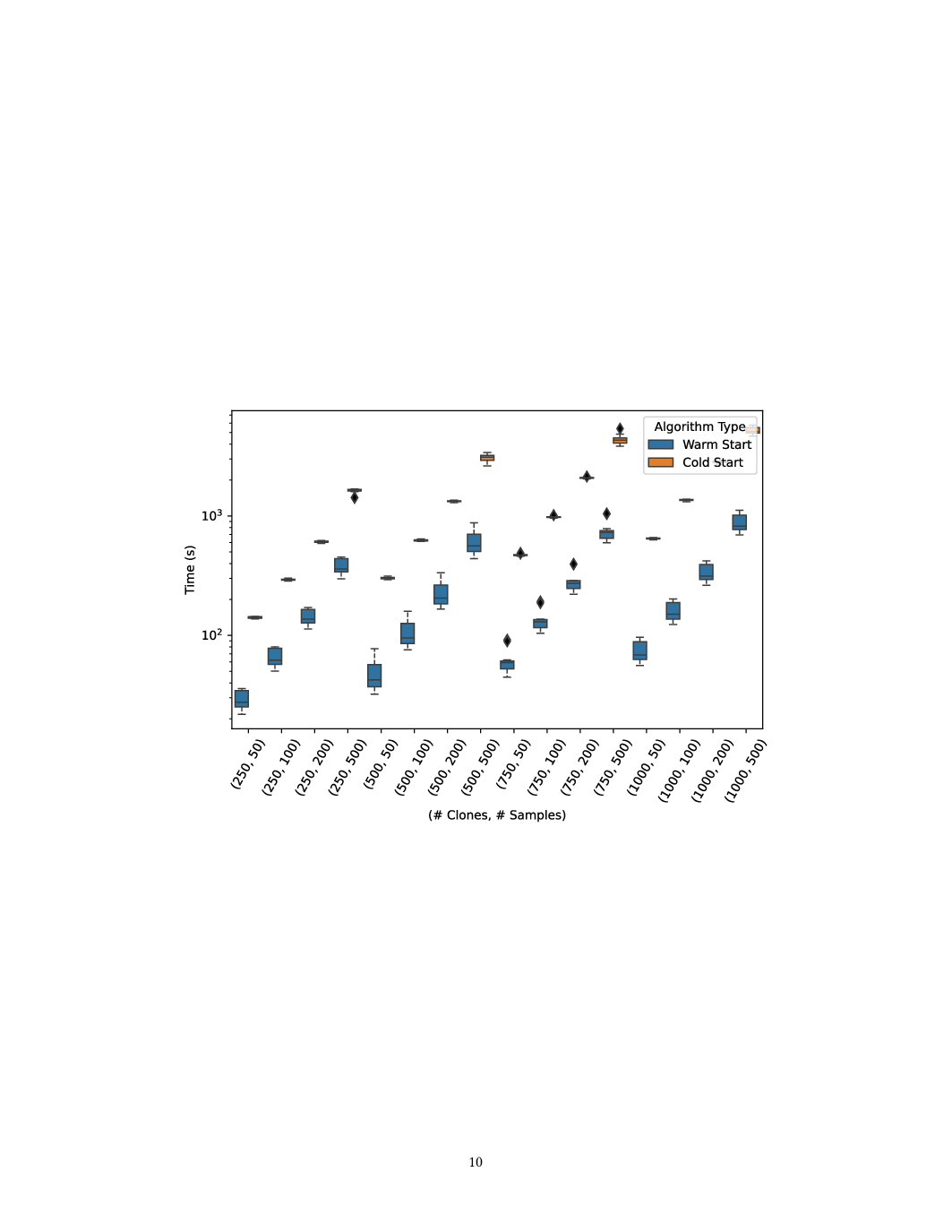

Supplement: S4 Fig — (TIFF) [file pcbi.1012631.s005.tiff]

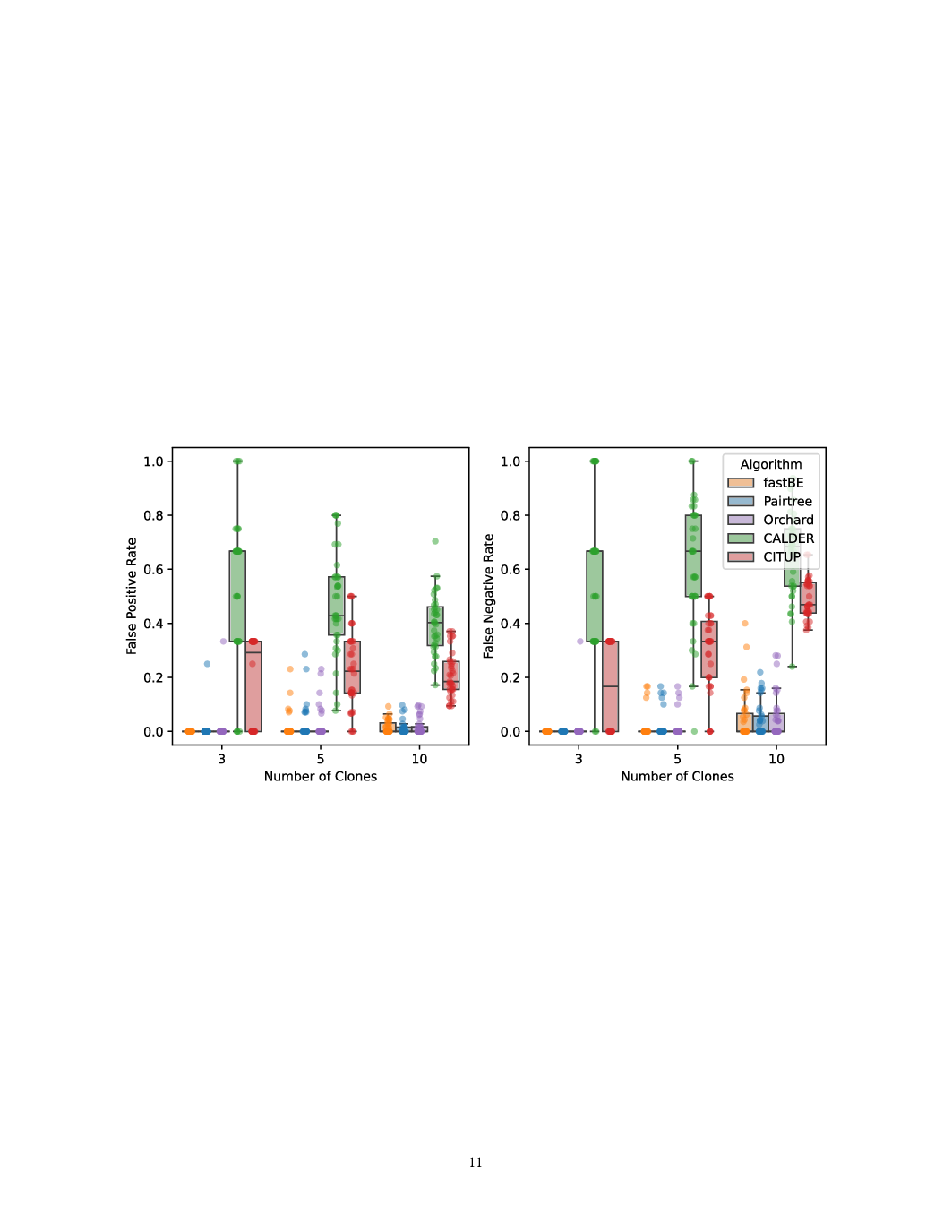

Supplement: S5 Fig — (TIFF) [file pcbi.1012631.s006.tiff]

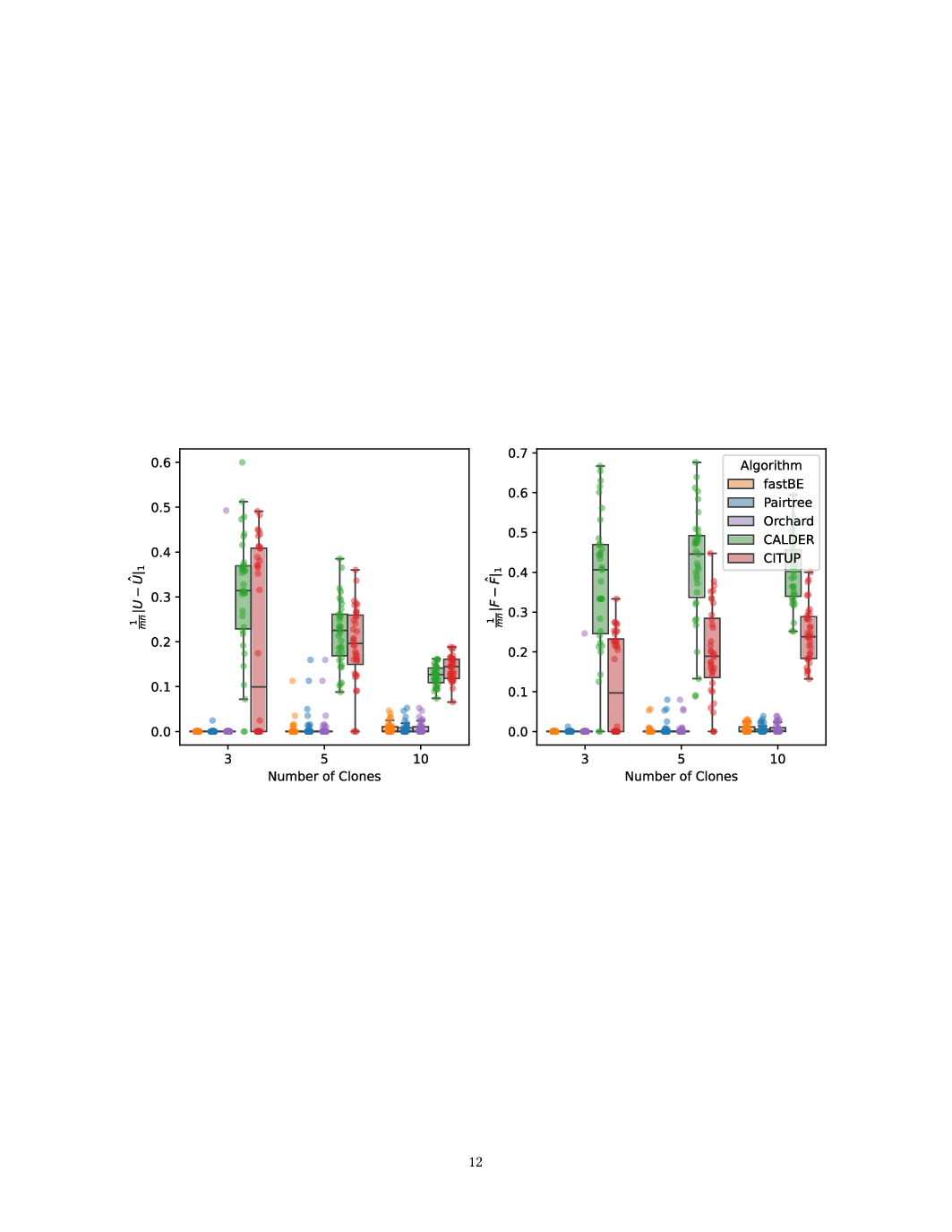

Supplement: S6 Fig — (TIFF) [file pcbi.1012631.s007.tiff]

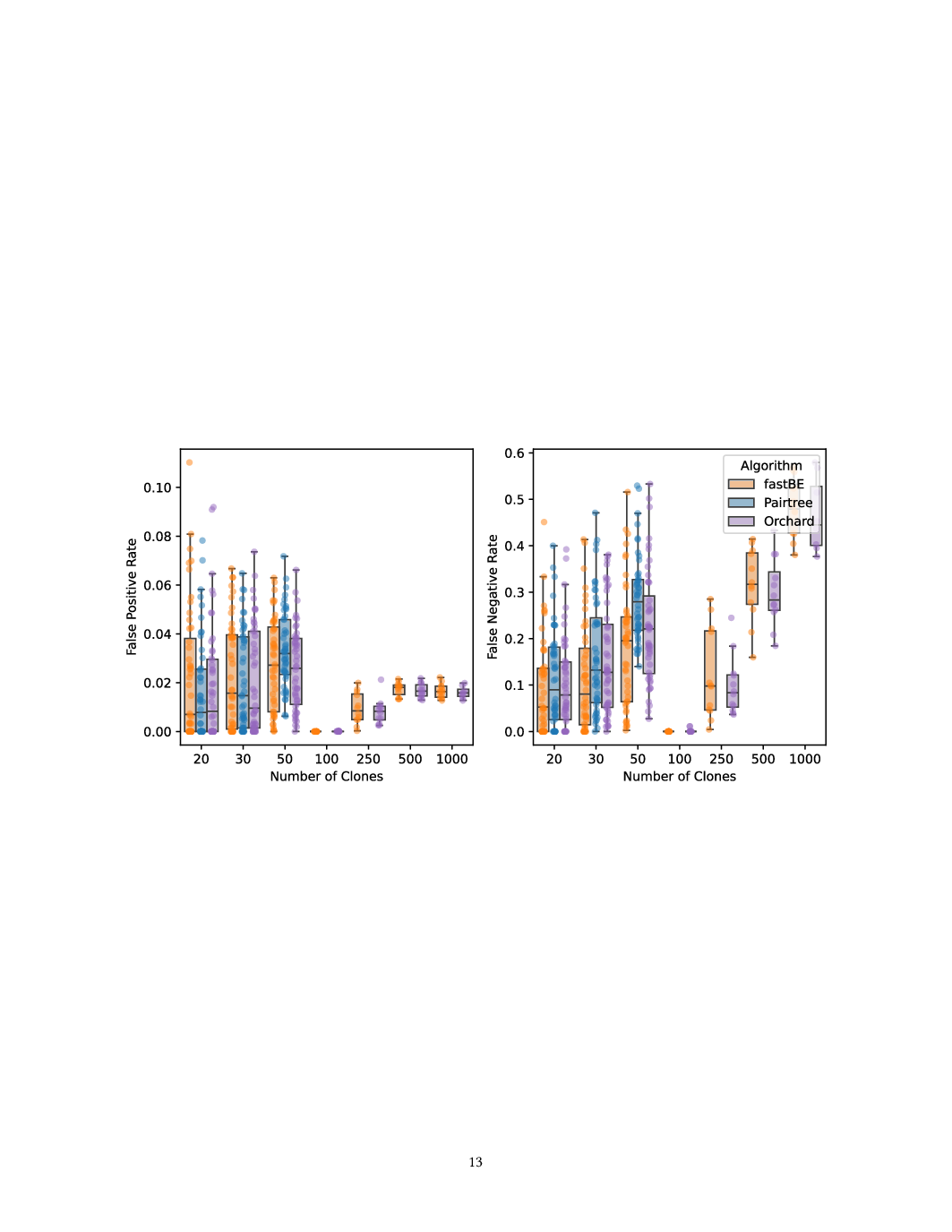

Supplement: S7 Fig — (TIFF) [file pcbi.1012631.s008.tiff]

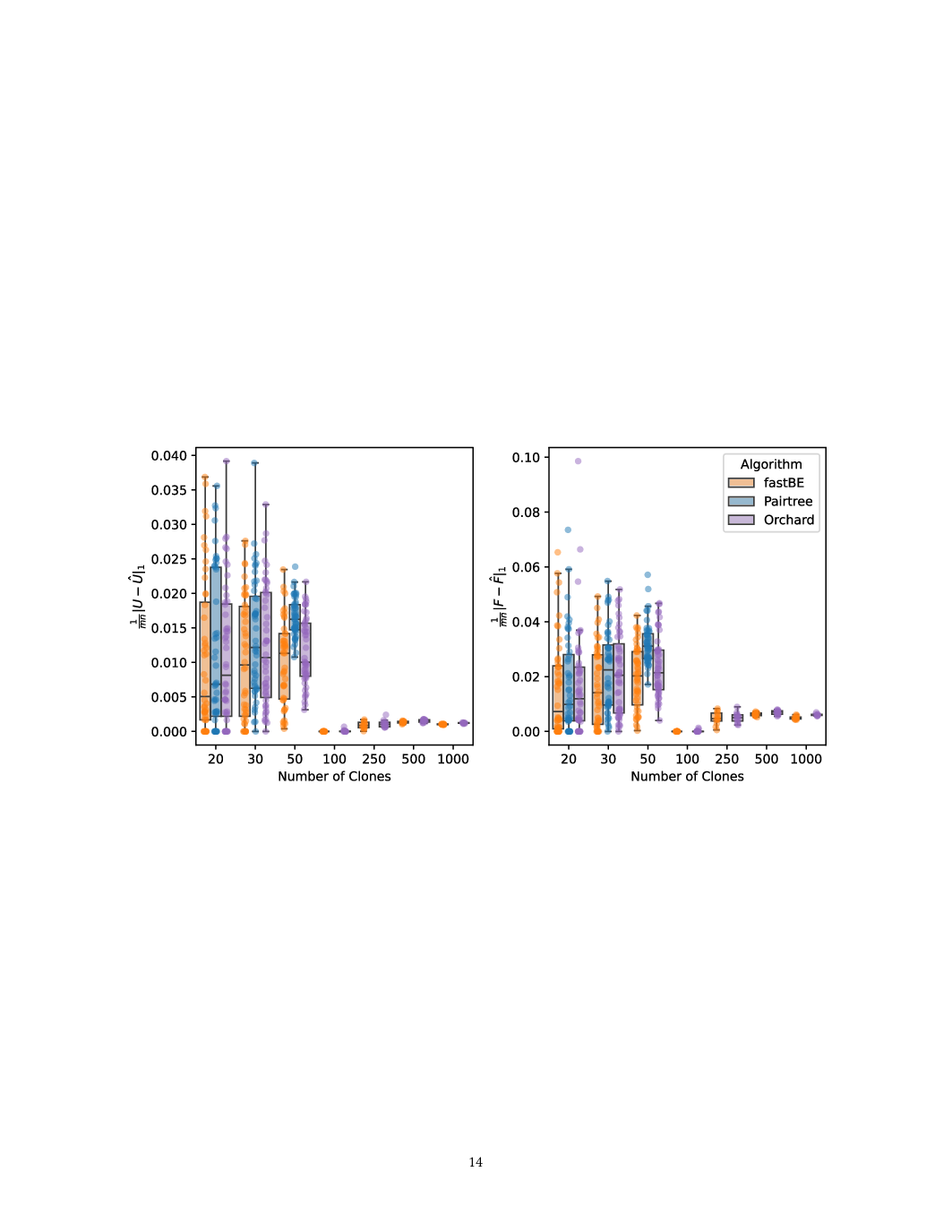

Supplement: S8 Fig — (TIFF) [file pcbi.1012631.s009.tiff]

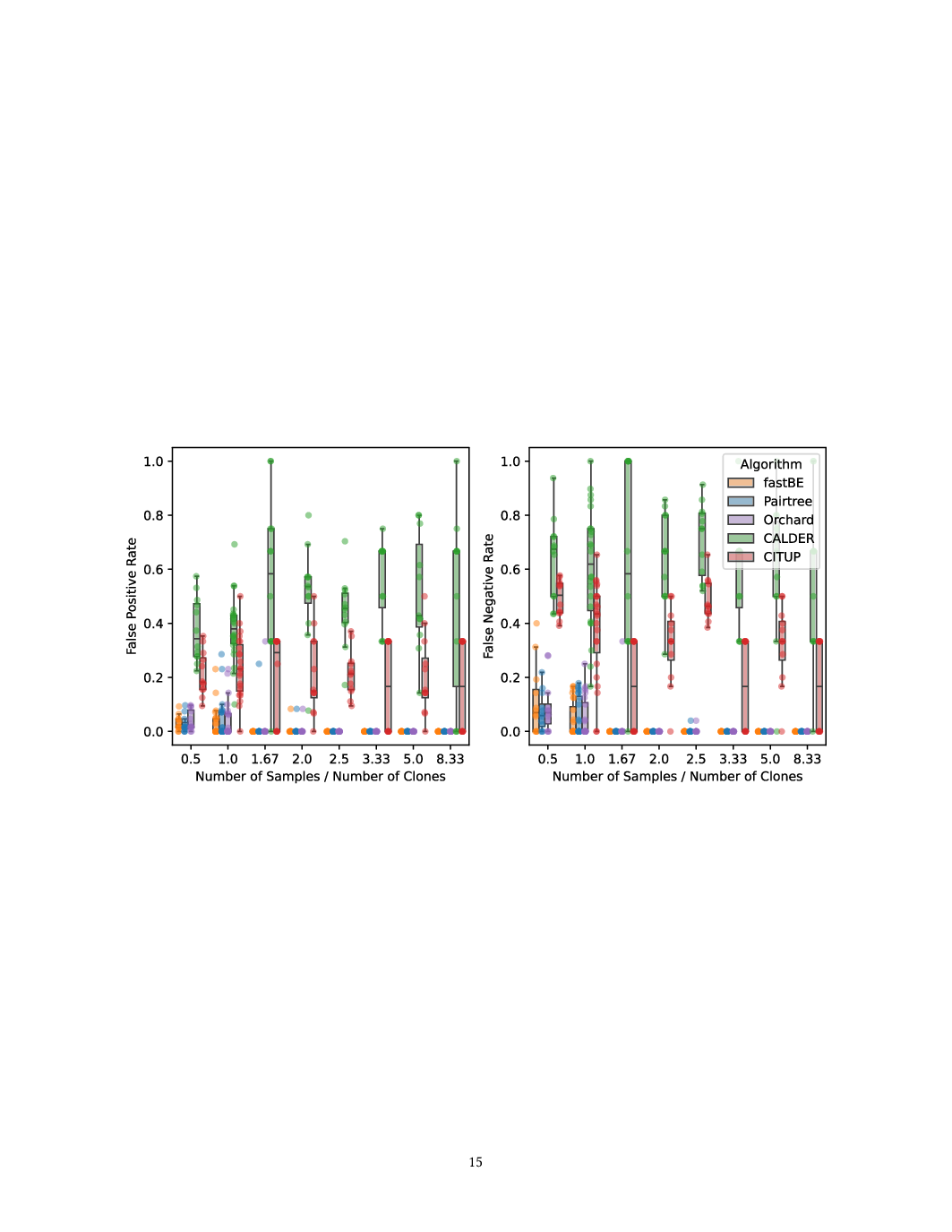

Supplement: S9 Fig — (TIFF) [file pcbi.1012631.s010.tiff]

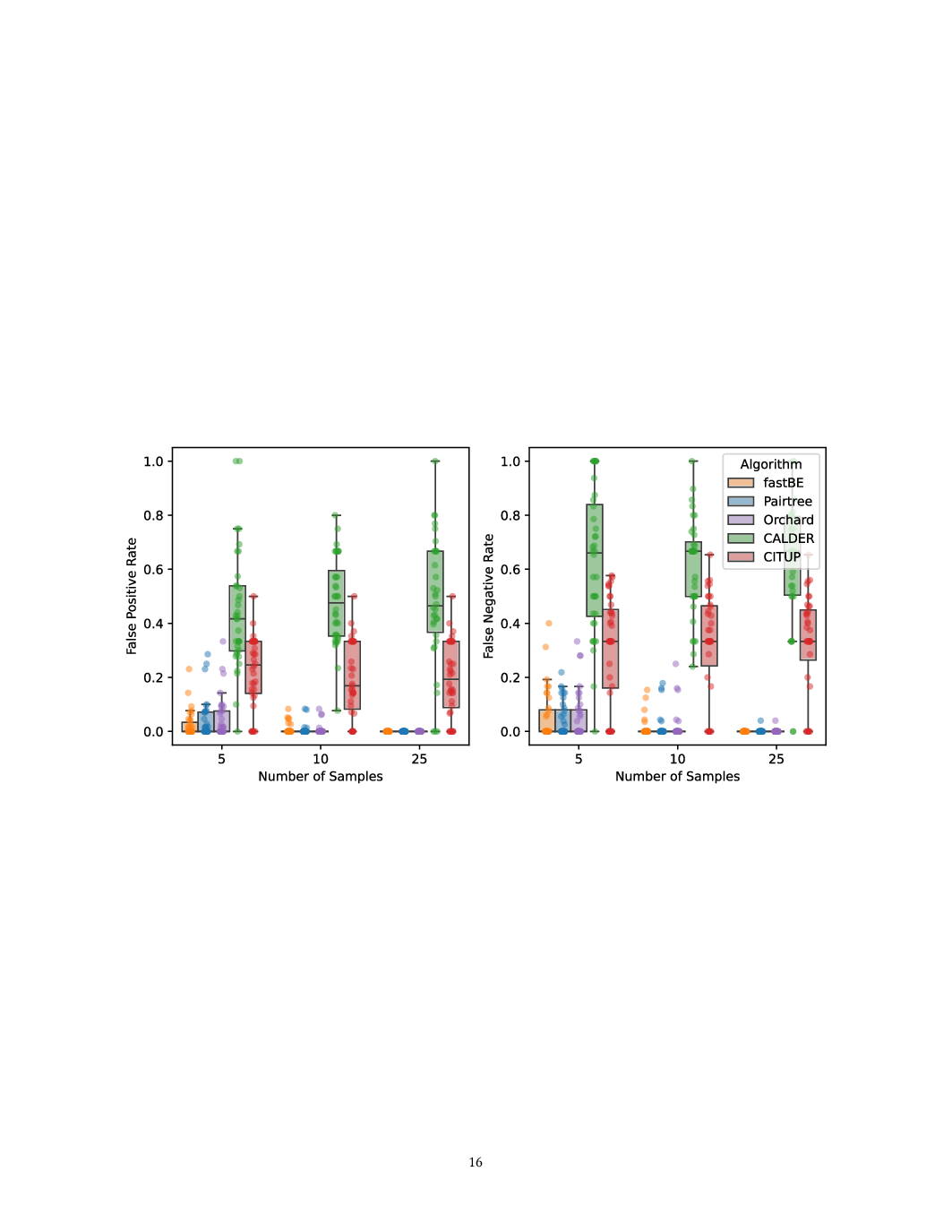

Supplement: S10 Fig — (TIFF) [file pcbi.1012631.s011.tiff]

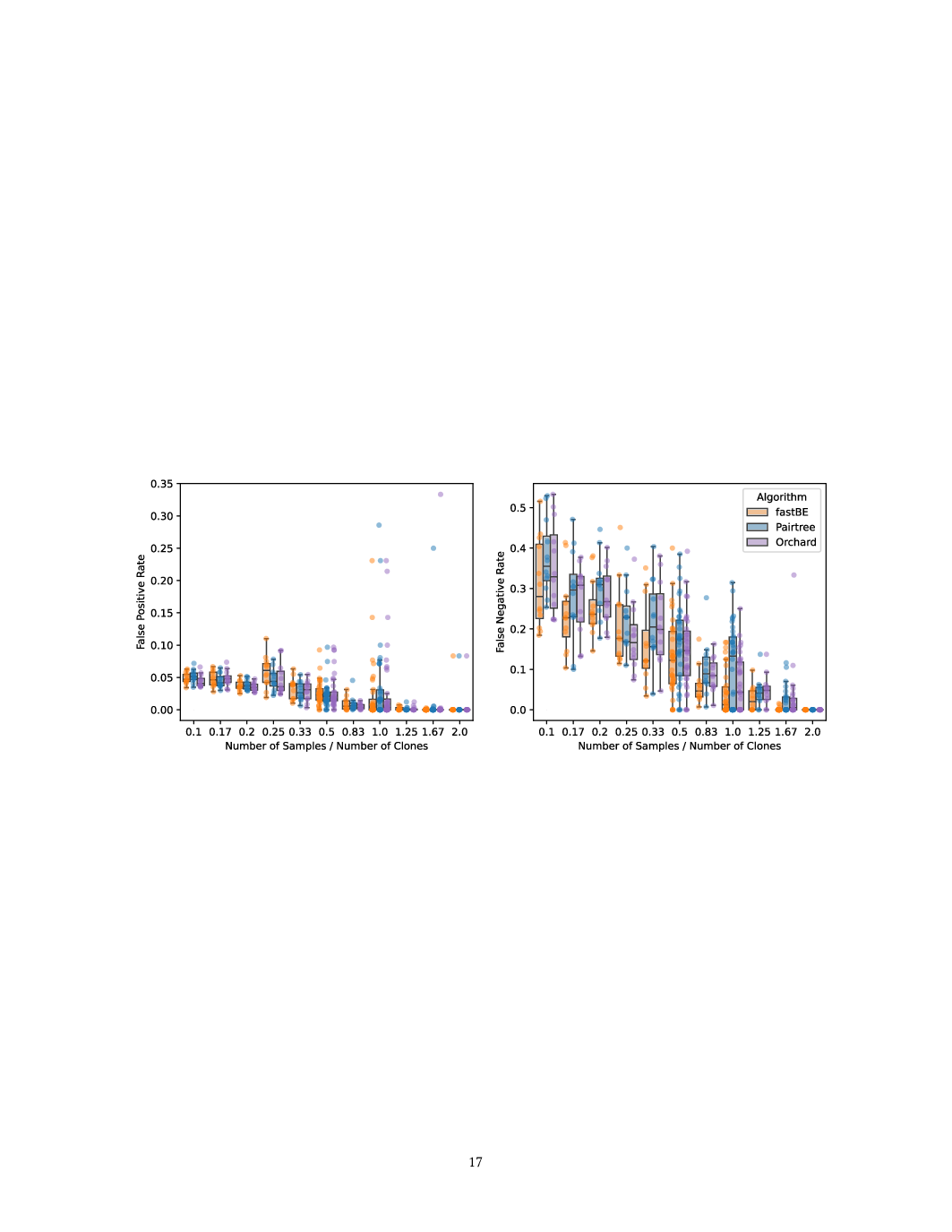

Supplement: S11 Fig — (TIFF) [file pcbi.1012631.s012.tiff]

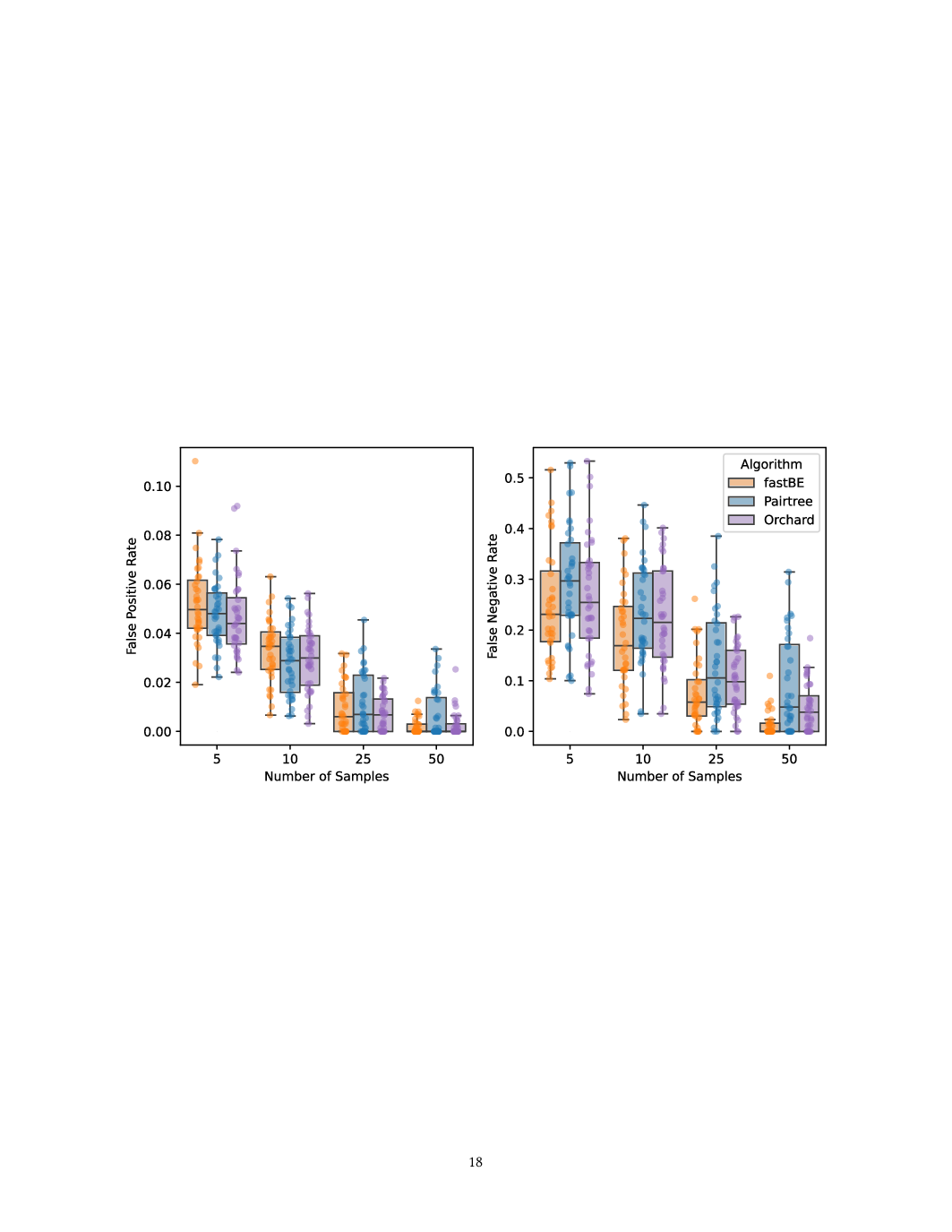

Supplement: S12 Fig — (TIFF) [file pcbi.1012631.s013.tiff]

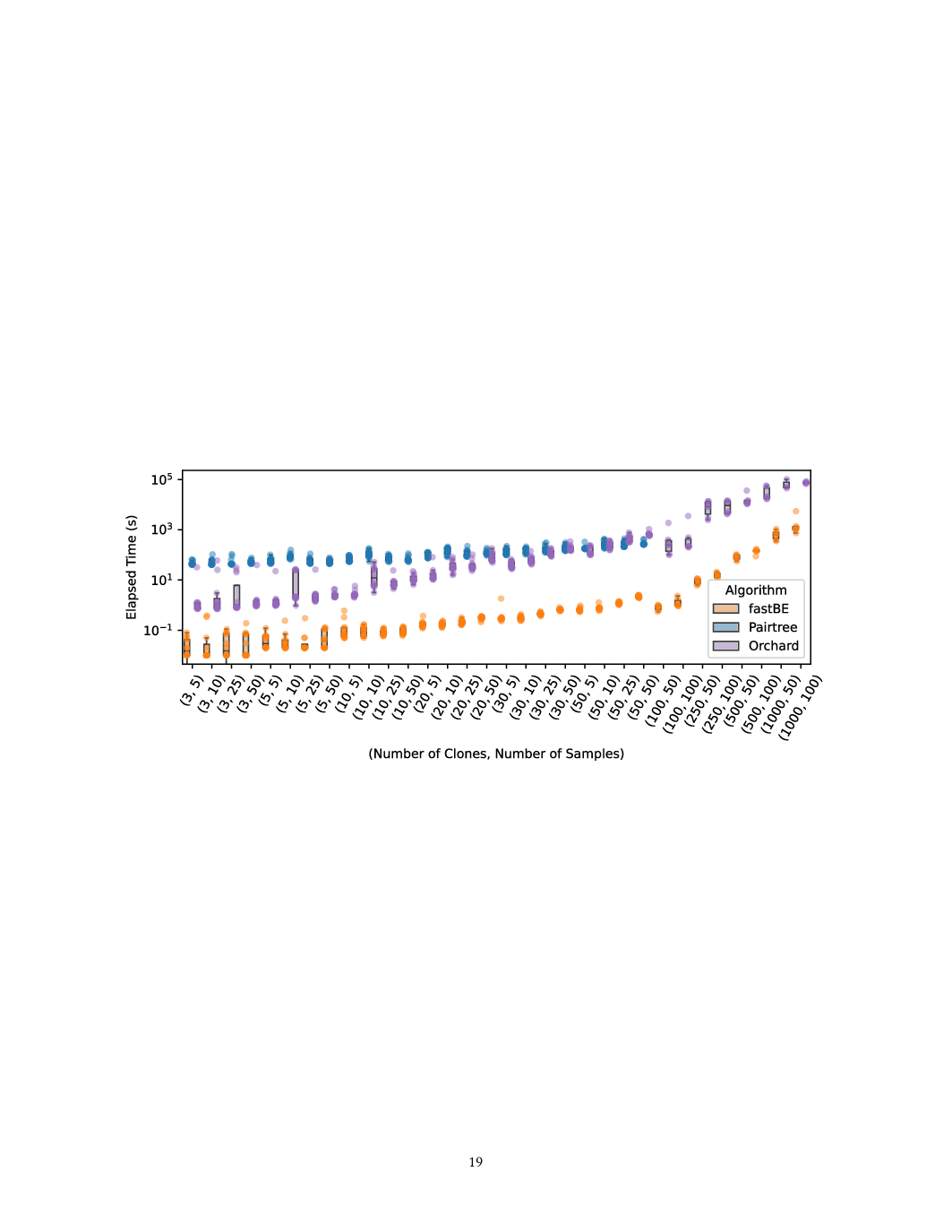

Supplement: S13 Fig — (TIFF) [file pcbi.1012631.s014.tiff]

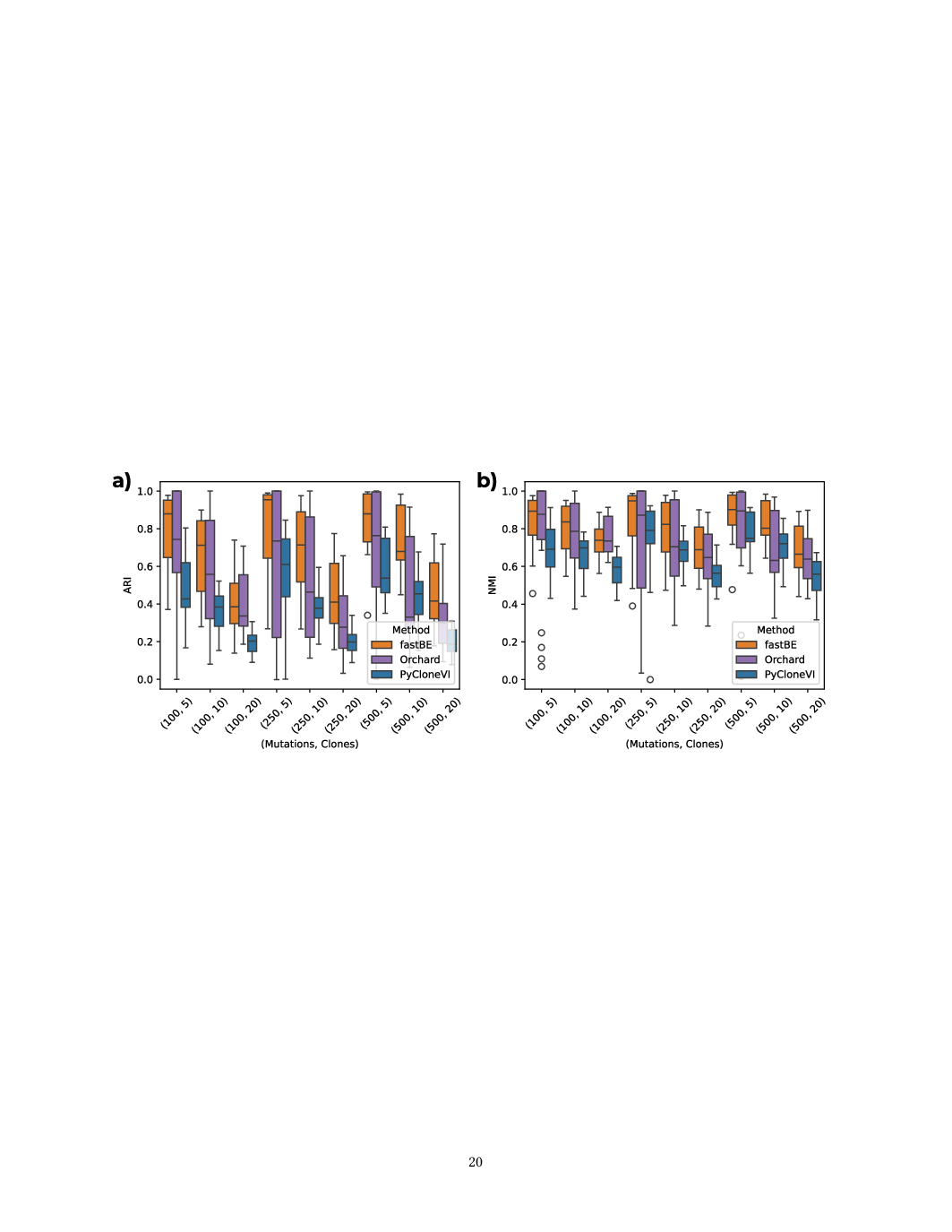

Supplement: S14 Fig — (TIFF) [file pcbi.1012631.s015.tiff]

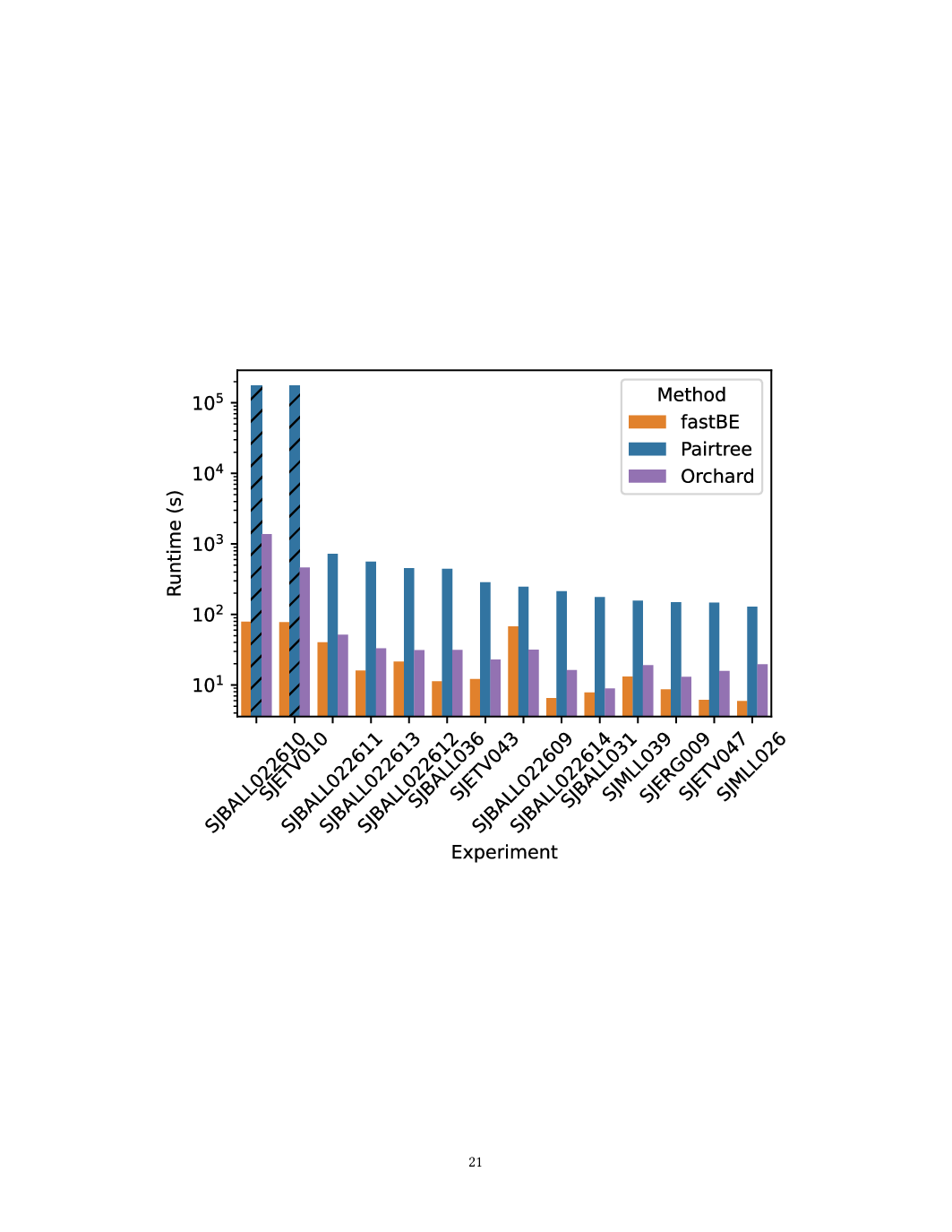

Supplement: S15 Fig — (TIFF) [file pcbi.1012631.s016.tiff]

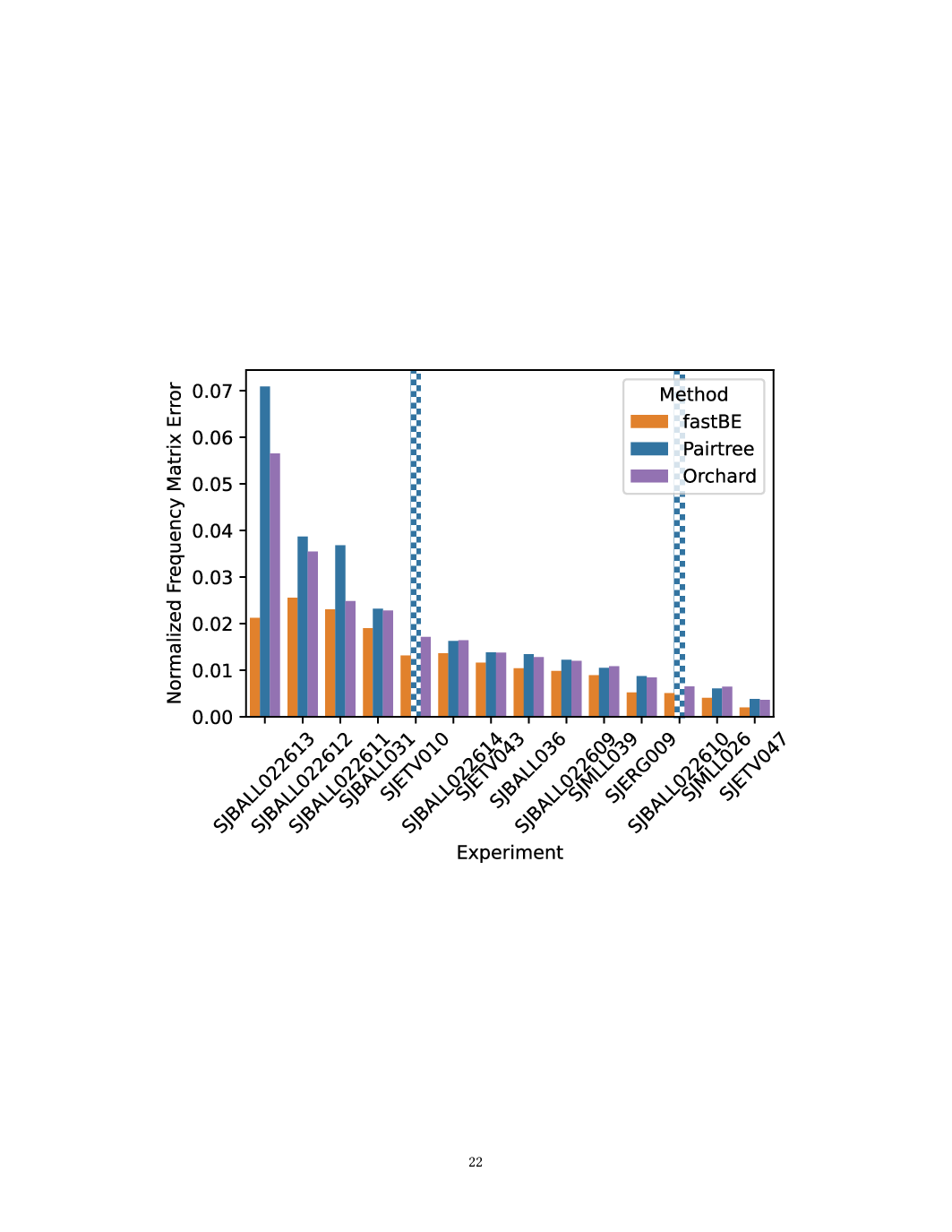

Supplement: S16 Fig — (TIFF) [file pcbi.1012631.s017.tiff]

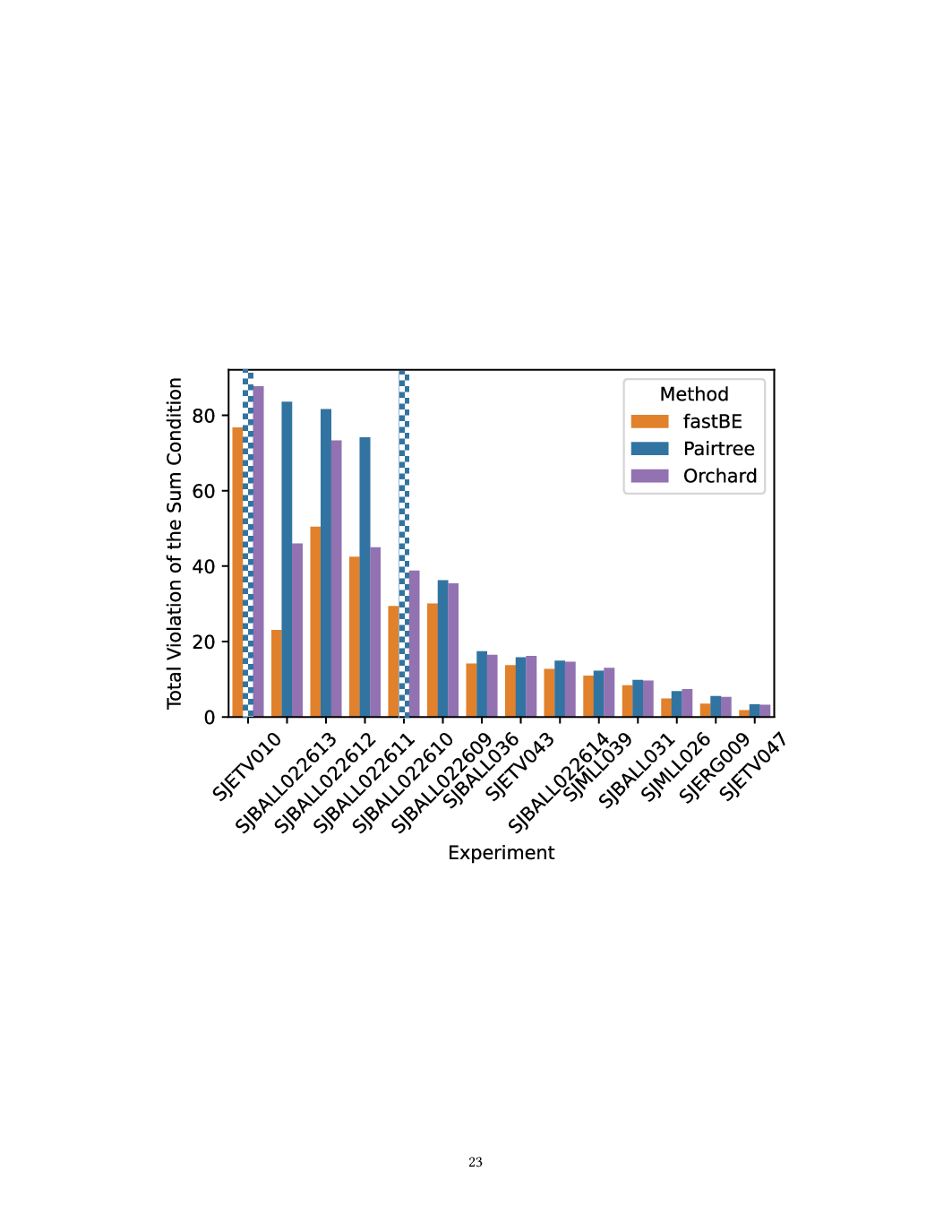

Supplement: S17 Fig — (TIFF) [file pcbi.1012631.s018.tiff]

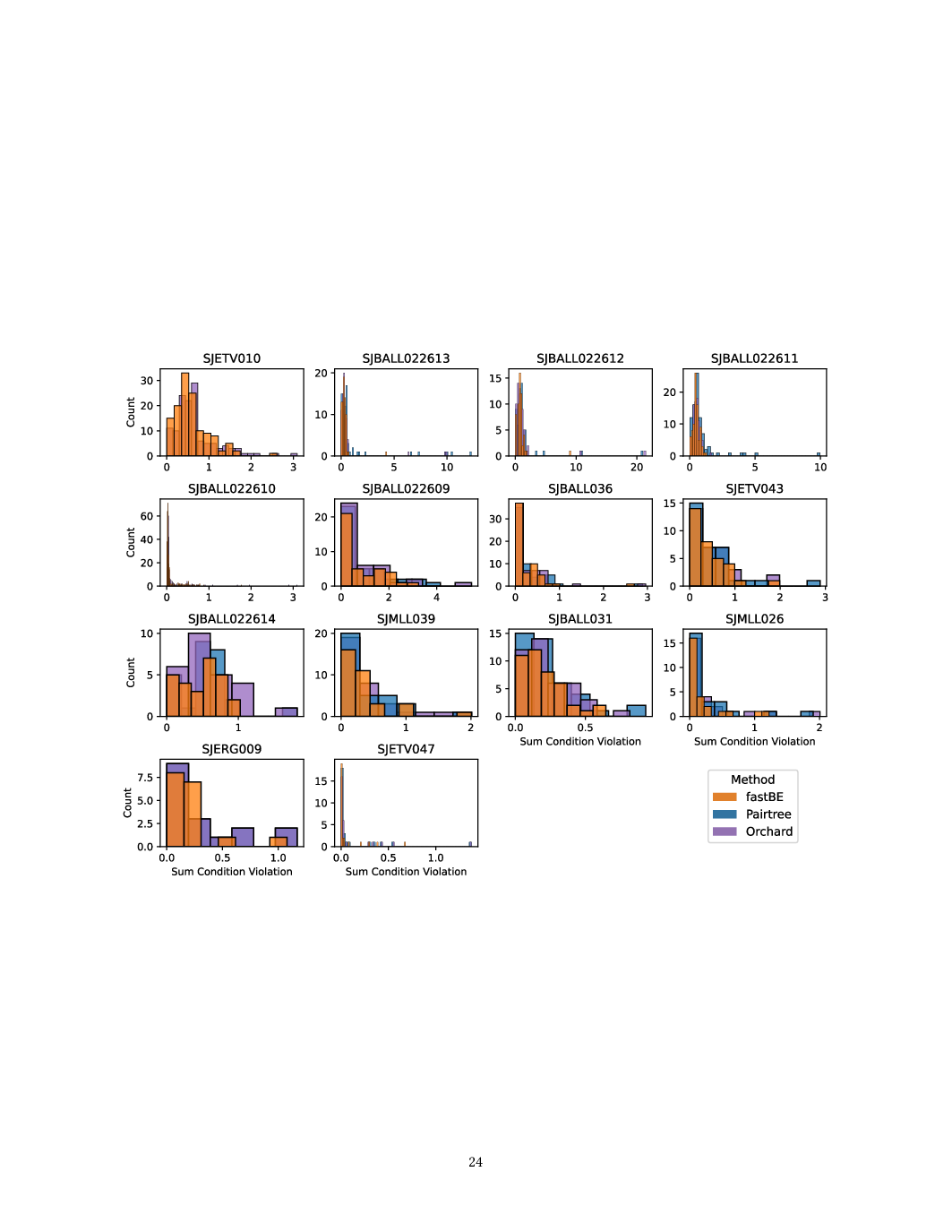

Supplement: S18 Fig — (TIFF) [file pcbi.1012631.s019.tiff]

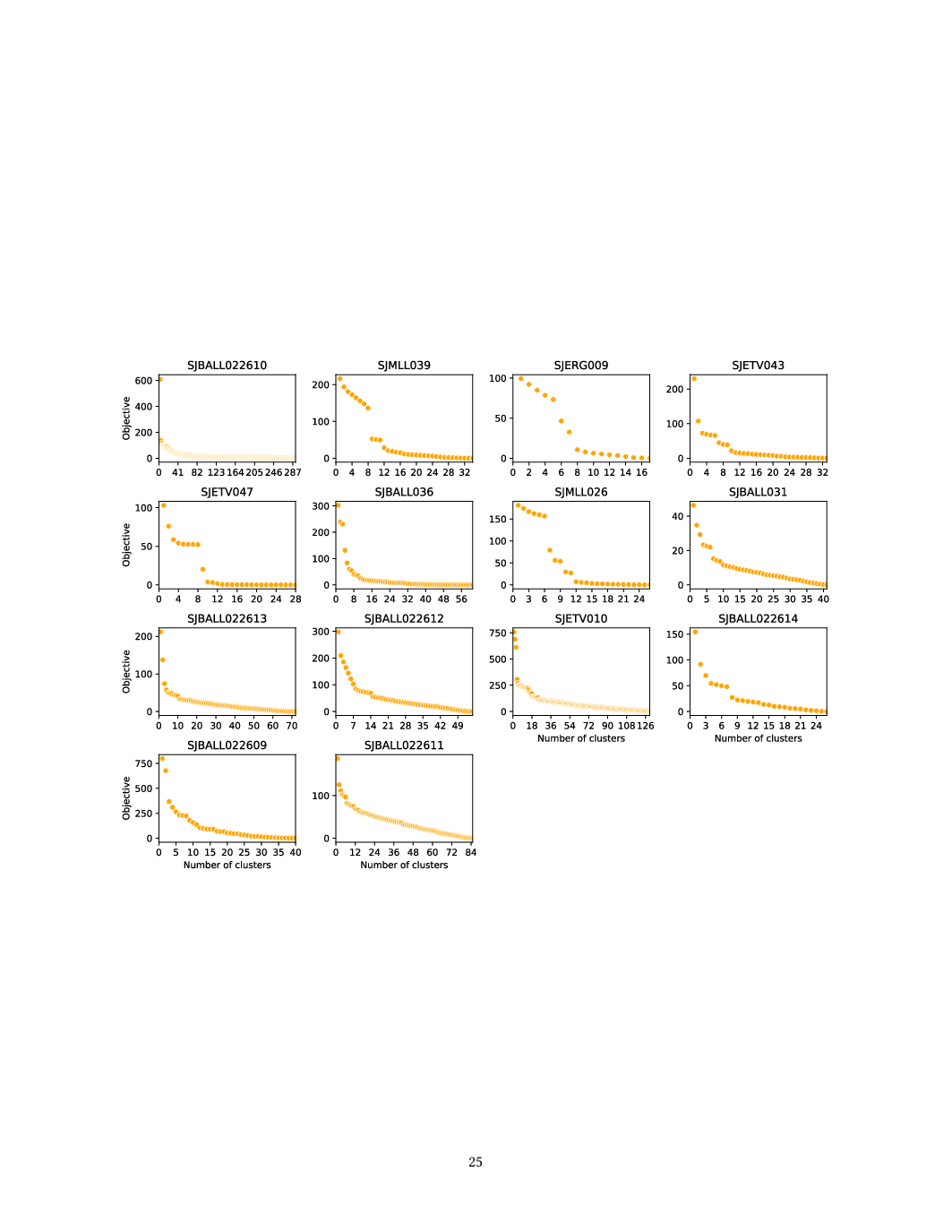

Supplement: S19 Fig — (TIFF) [file pcbi.1012631.s020.tiff]

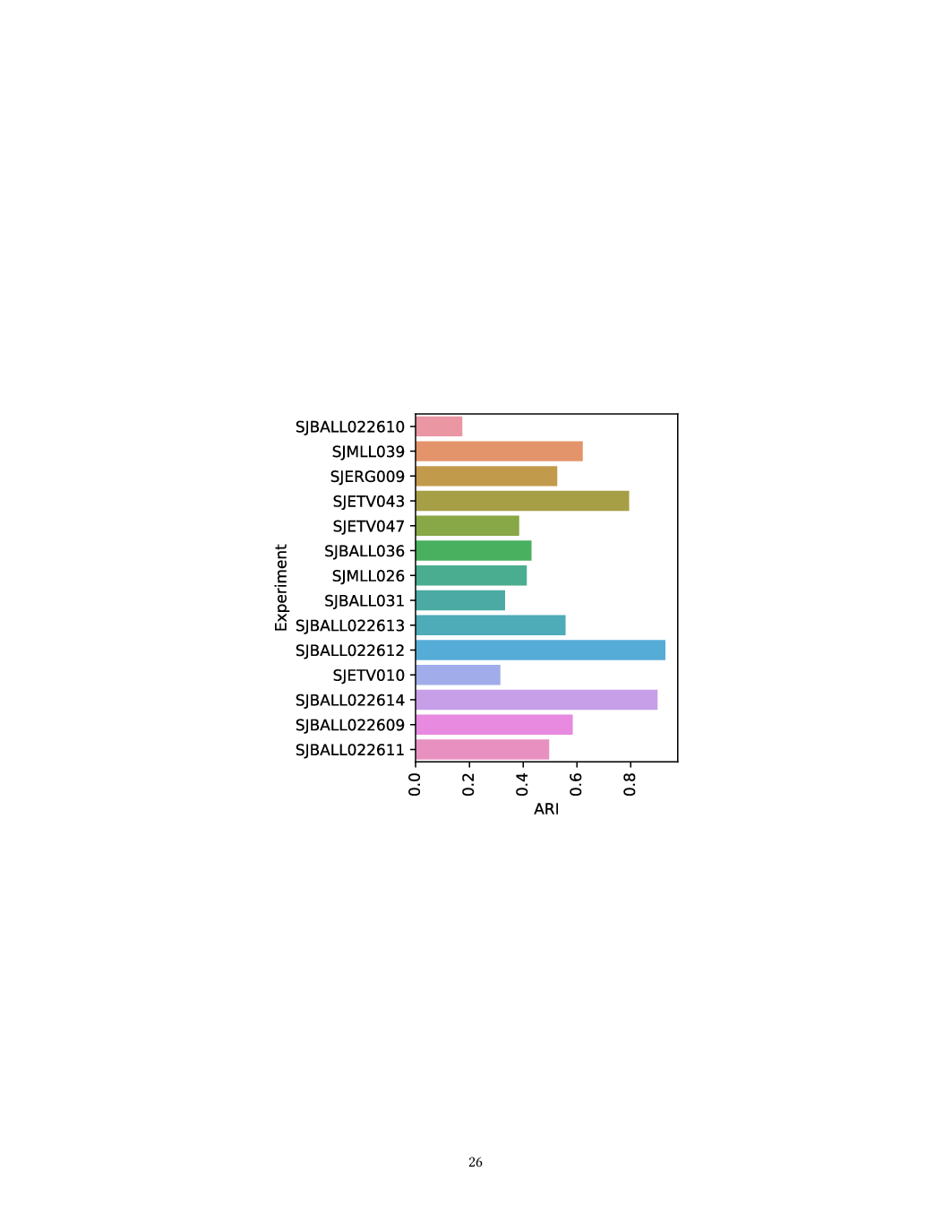

Supplement: S20 Fig — (TIFF) [file pcbi.1012631.s021.tiff]

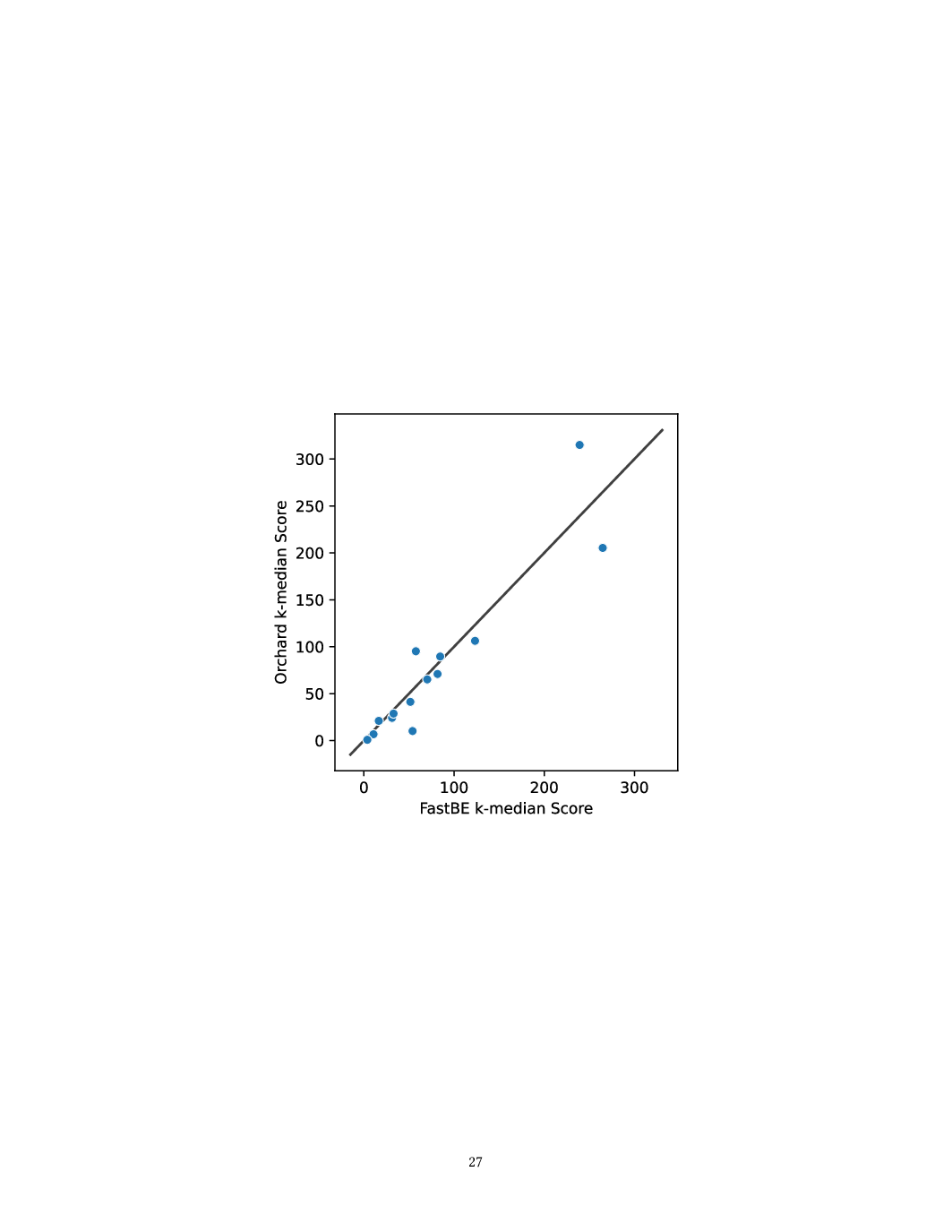

Supplement: S21 Fig — (TIFF) [file pcbi.1012631.s022.tiff]

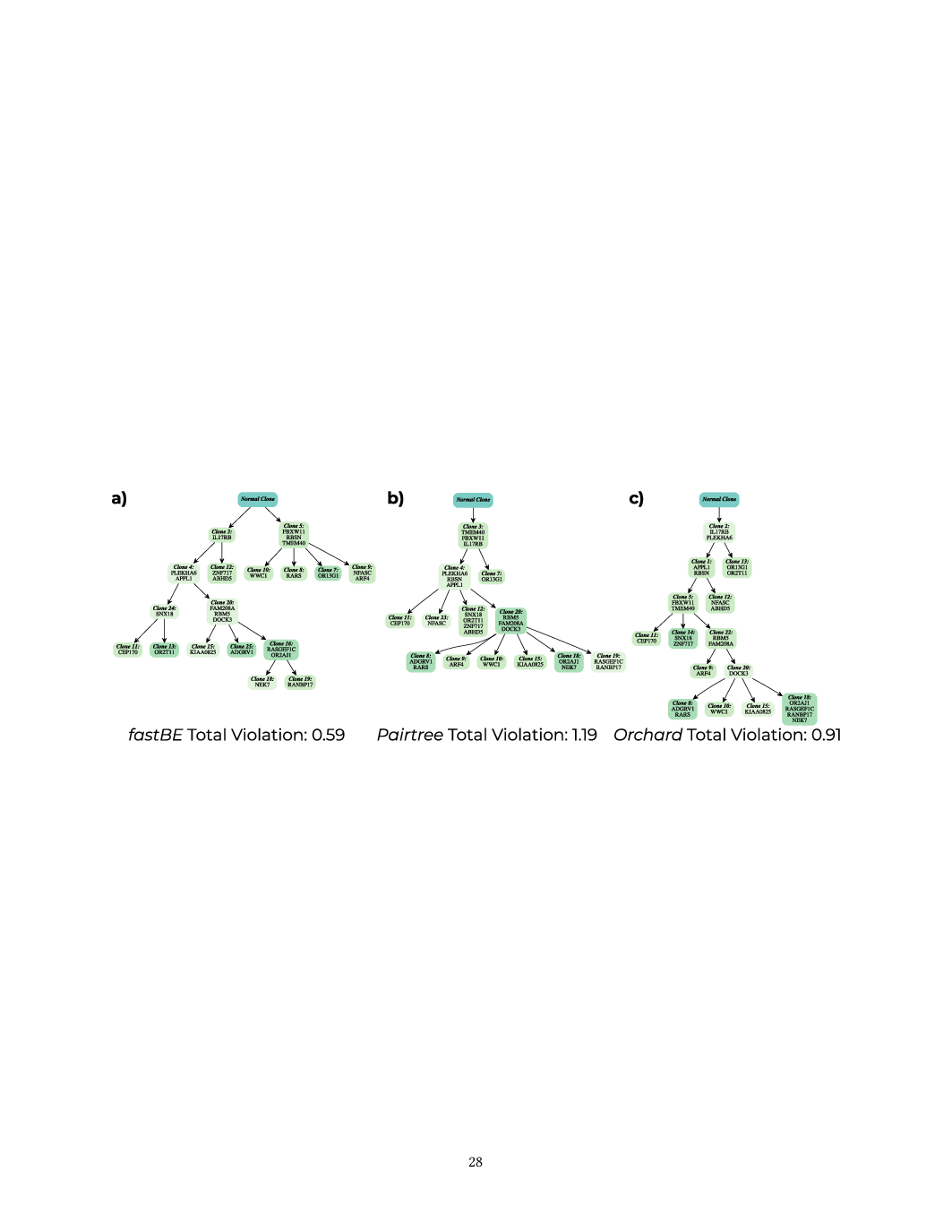

Supplement: S22 Fig — (TIFF) [file pcbi.1012631.s023.tiff]

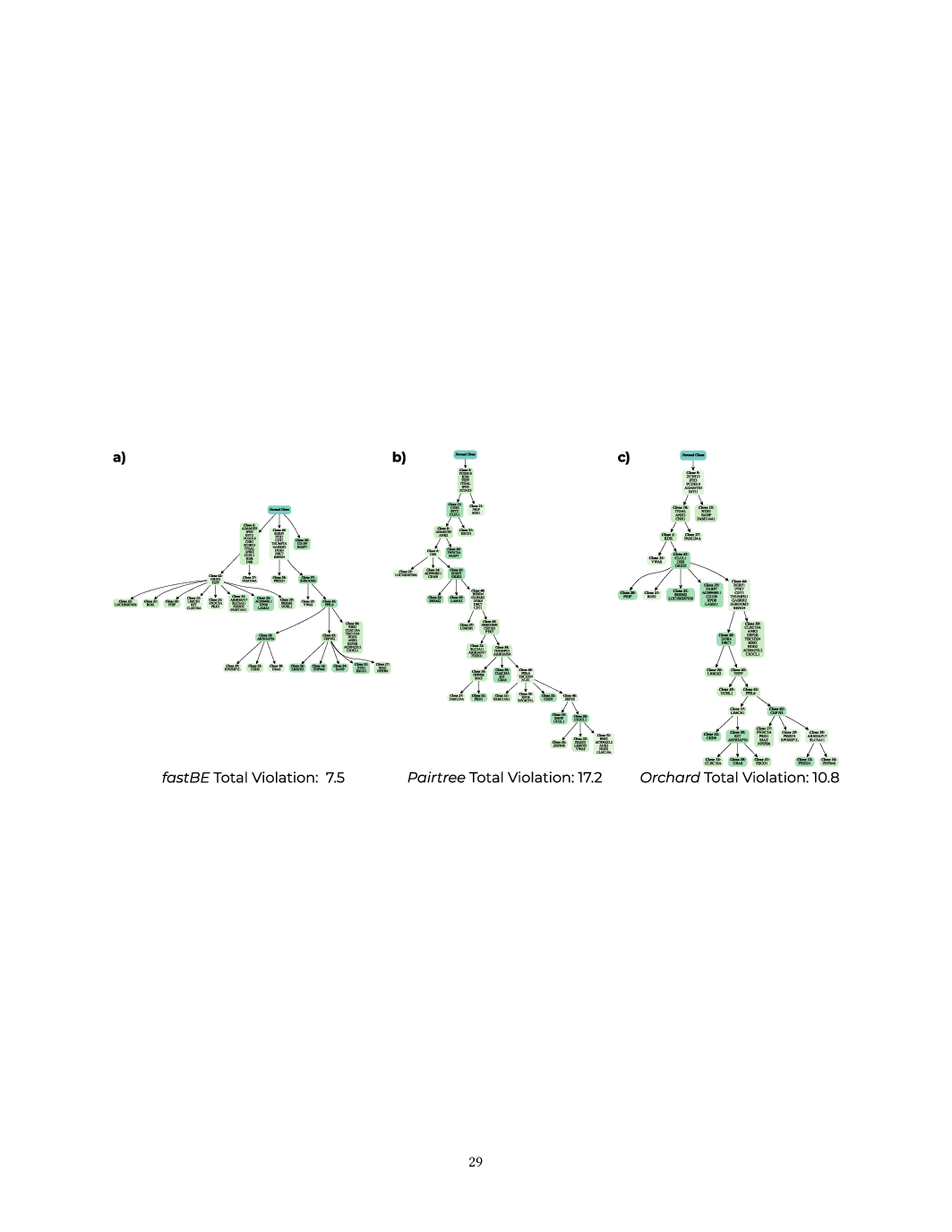

Supplement: S23 Fig — (TIFF) [file pcbi.1012631.s024.tiff]

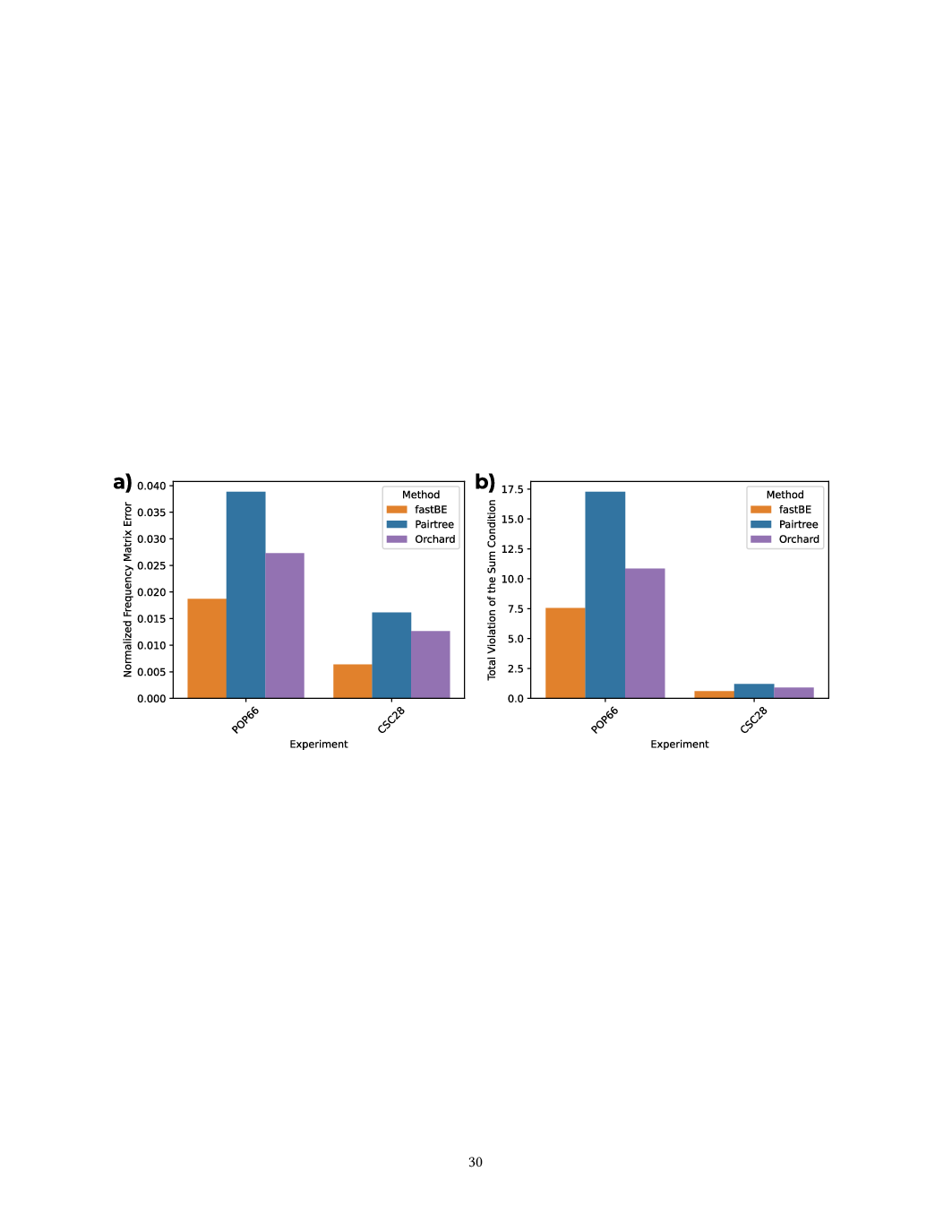

Supplement: S24 Fig — (TIFF) [file pcbi.1012631.s025.tiff]

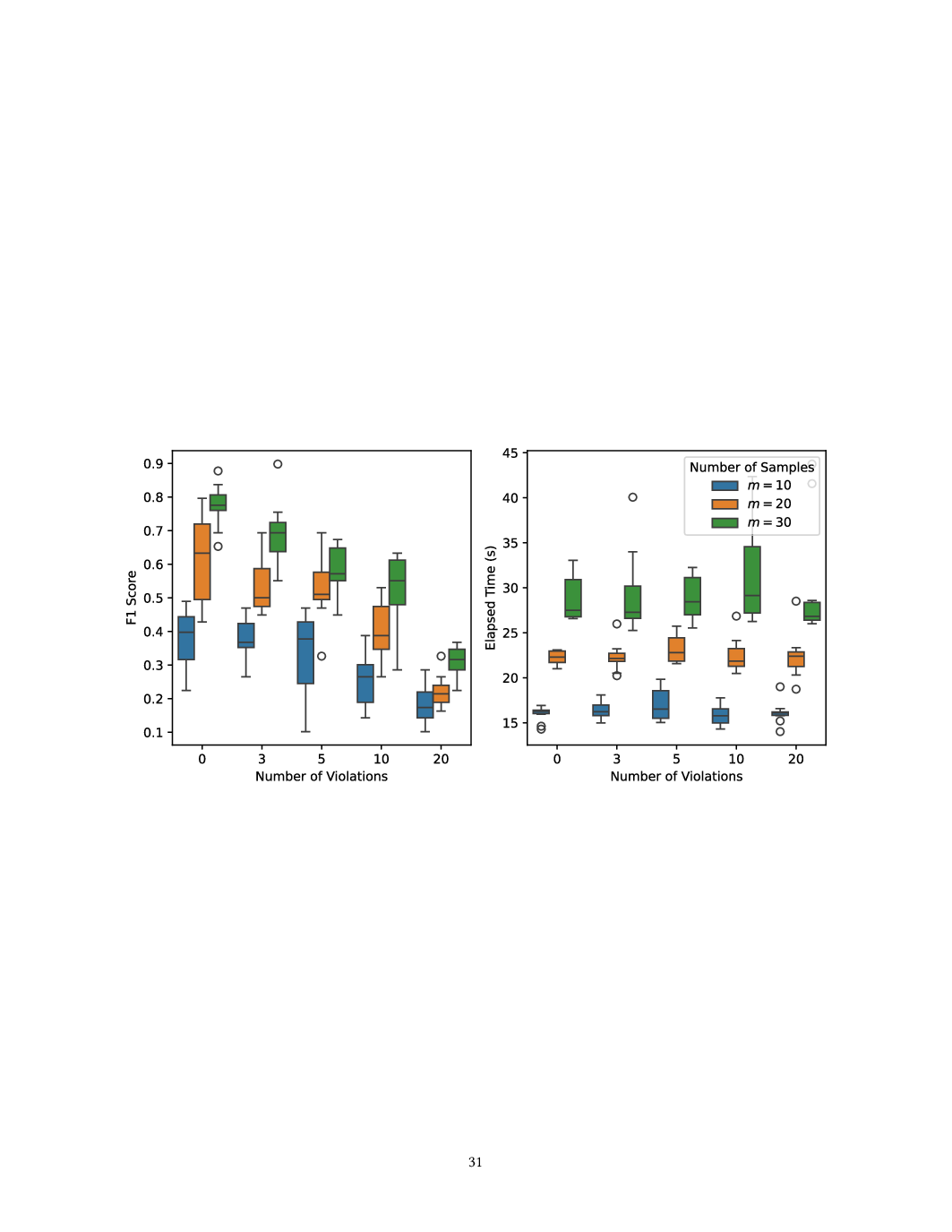

Supplement: S25 Fig — (TIFF) [file pcbi.1012631.s026.tiff]
